# Methods for meta-analysis and meta-regression of binomial data: concepts and tutorial with Stata command metapreg

**DOI:** 10.1186/s13690-023-01215-y

**Published:** 2024-01-29

**Authors:** Victoria Nyawira Nyaga, Marc Arbyn

**Affiliations:** Unit of Cancer Epidemiology, Sciensano, Brussels, Belgium

**Keywords:** Meta-analysis, Meta-regression, Network meta-analysis, Stata, Logistic regression, Binomial

## Abstract

**Background:**

Despite the widespread interest in meta-analysis of proportions, its rationale, certain theoretical and methodological concepts are poorly understood. The generalized linear models framework is well-established and provides a natural and optimal model for meta-analysis, network meta-analysis, and meta-regression of proportions. Nonetheless, generic methods for meta-analysis of proportions based on the approximation to the normal distribution continue to dominate.

**Methods:**

We developed metapreg, a tool with advanced statistical procedures to perform a meta-analysis, network meta-analysis, and meta-regression of binomial proportions in Stata using binomial, logistic and logistic-normal models. First, we explain the rationale and concepts essential in understanding statistical methods for meta-analysis of binomial proportions and describe the models implemented in metapreg. We then describe and demonstrate the models in metapreg using data from seven published meta-analyses. We also conducted a simulation study to compare the performance of metapreg estimators with the existing estimators of the population-averaged proportion in metaprop and metan under a broad range of conditions including, high over-dispersion and small meta-analysis.

**Conclusion:**

metapreg is a flexible, robust and user-friendly tool employing a rigorous approach to evidence synthesis of binomial data that makes the most efficient use of all available data and does not require ad-hoc continuity correction or data imputation. We expect its use to yield higher-quality meta-analysis of binomial proportions.

**Supplementary Information:**

The online version contains supplementary material available at 10.1186/s13690-023-01215-y.

**Table:** 

**Text box 1. Contribution to the literature**	
$\bullet$ Explain the key concepts and rationale in methods for meta-analysis.	
$\bullet$ Highlight the misconceptions, theoretical and methodological flaws in the current methods for meta-analysis of proportions.	
$\bullet$ Explain the logistic regression models employed by the Stata package metapreg for meta-analysis, network meta-analysis, and meta-regression of proportions.	
$\bullet$ Demonstrate metapreg’s functionality using data from previously published meta-analyses.	
$\bullet$ Conduct a simulation study to compare metapreg’s performance with current methods under a broad range of conditions including high over-dispersion and small meta-analysis.	

## Background

Meta-analyses offer an efficient way to synthesize information from different sources, facilitating evidence-based decision-making. Unless sound statistical techniques are used, inference from a poorly conducted meta-analysis can lead to erroneous conclusions.

Meta-analysis is often viewed as a method for aggregating study results into a single estimate [1]. However, it is a study of multiple studies, aiming to synthesize their findings [2, 3]. Most techniques employed in practice for combining study results are grounded in either the common-effect (CE, also known as the fixed-effect) model or the random-effects (RE) model. These models are underpinned by distinct underlying assumptions about the included studies and their summary results are subject to differing interpretations [4, 5].

We distinguish the two models by their mathematical expressions, statistical properties and practical difference. Traditionally, a linear regression model is employed to directly fit study parameter estimates, simplifying the statistical aspects with straightforward equations known as weighted least squares (WLS). In the CE model, the observed effect in a study (denoted as ‘j’) is expressed as the sum of a fixed effect $\theta$ common to all studies and a sampling error, represented by the estimated within-study variance $\hat{\nu }_j^2$. This formulation implies that the observed variability in the data is solely attributed to chance. However, it is common to find that the observed variability exceeds what can be explained by the CE model. This is referred to as overdispersion. Neglecting this excess variation results in an underestimation of the standard error of the estimate for $\theta$, leading to potentially misleading inferences. To address overdispersion, the standard approach introduces a study-specific random effect $\delta _j$ with a distribution $N(0, \tau ^2)$ into the CE model to account for the excess variation. This is the RE model. $\tau ^2$ represents the between-study variation. When $\tau ^2$ equals zero, the RE model collapses into the CE model. In practical application, techniques based on the RE model typically do not directly address the issue of overdispersion. Instead, they employ a strategy to alleviate its effects by adjusting the study variance with the incorporation of the $\tau ^2$ estimate. Consequently, this adaptation results in an enlargement of the standard error associated with the RE model’s $\theta$ estimate. In an alternative approach, overdispersion is considered a nuisance and corrects the standard errors of the CE model’s $\theta$ estimate.

A meta-analysis of proportions makes inferences about the study parameter $\pi$ given the number of study events *n* and the sample size *N* of the study. Naturally, *n* is assumed to follow a binomial distribution and functions like the odds ratio (OR) and/or the rate ratio (RR) are derivatives of $\pi$. The ordinary logistic regression model is a generalized linear model (GLM) [6]; an extension of the linear regression model for binomial data. When the observed variation is more than explained by the ordinary logistic regression model, normally distributed error terms are added to the model corresponding to different sources of variation in the data. The resulting logistic regression model formulation is a generalized linear mixed model(GLMM) [6, 7]. The alternative approach to handle over-dispersion in the binomial distribution uses the beta-binomial regression model. This is the exact likelihood framework for meta-analysis of proportions. The computations involved in these models are intensive and processing the model parameter estimates into meaningful results requires programming and statistical expertise.

To simplify the problem, the study estimate $\hat{\pi } = \frac{n}{N}$ (or a function of $\hat{\pi }$) and its variance are plugged in the WLS computations of the linear regression model. This is the approximate likelihood framework where the the normal distribution approximates the distribution of a binomial parameter. Stata packages developed within this framework include metaprop [8], metan [9], metaan [10] and mvmeta [11]. As from Stata 16, the CE and RE models can be fitted using the command meta [12].

WLS is an extension of the ordinary least squares (OLS). The computations in OLS count the data points from each study equally. Conversely, WLS counts more the data points from studies with low error variances by giving them more weight - the inverse variance (IV) weighting scheme. The most popular procedure for calculating the weights in the RE model was proposed by Dersimonian-Liard (DL) [13] - a parameter $\tau ^2$ is added to the variance of the study parameter estimates. To bridge the statistical principles of the CE linear regression model and the Dersimonian-Liard RE model, Doi et al. [1, 14] proposed the inverse variance heterogeneity (IVhet) and the quality-effects (QE) models classified in the quasi-likelihood framework. The Stata procedures metan [9] and mvmeta [11] extend to this framework.

There is a misconception that methods for meta-analysis that explicitly define weights are sound. However, treating the within-study standard errors used in weighting the studies as known is a fundamental flaw [15]. A major criticism towards the current RE linear model for meta-analysis is its use of study weights that are not inversely proportional to the study sizes [16]. The logistic regression is well-established and provide a natural and optimal model for evidence synthesis of binomial data. However, their rationale, certain theoretical and methodological aspects are poorly understood especially their unorthodox implicit weighting mechanism. The logistic regression estimates are the limit of a sequence of WLS where the weight changes at each cycle. Throughout the optimization process, studies with more statistical power get more weight.

With the availability of software for maximum likelihood (ML) estimation of model parameters within the exact likelihood framework, the computational simplicity of the WLS is no longer relevant. metaprop_one [17] is a Stata procedure developed in 2014 within the exact likelihood framework. However, it has limited capabilities and functionality. It synthesizes results from one group or per subgroup by one categorical covariate. Moreover, the model parameter estimates are only partially processed for useful inference. To further close the gap between accessible procedures for meta-analysis of proportions and the end-users, we developed metapreg [18] in Stata 14 to perform meta-analysis, meta-regression and network meta-analysis of binomial proportions using binomial, logistic and logistic-normal models.

The rest of the paper is as follows. First, we establish the connection from classical regression model to meta-analysis. This will be followed by a quick review on the current methods for meta-analysis. We then discuss the theoretical and methodological flaws in the current methods for meta-analysis of proportions. We will then describe the logistic regression models for meta-analysis of proportions and demonstrate the fitting of the models with metapreg using data from previously published meta-analyses. Afterward, we will show that the RE logistic regression model is robust under a broad range of conditions including high over-dispersion and small meta-analysis. The last section concludes with a discussion.

## The classical linear model

The classic linear regression model is a particular case of the GLM. From a statistical point of view, a model is a mathematical expression formulated to decently describe the behavior of *I* outcome responses of a variable $Y = (Y_1, \ldots , Y_I)$ and the covariates $X = (X_1, \ldots , X_k)$ in a given study.

### Formulation

A linear regression model expresses the statistical relation between the outcome responses and the covariates as the sum of two components; the mean function (expressed in terms of the covariates) and the error function

1 $$\begin{aligned} Y_i = \text {mean function} + \text {error}_i\ \text {for i} = 1, \ldots ,I \end{aligned}$$

where $Y_i$ denotes the $i^{th}$ data point. Let $X_0 = 1$. In a simple linear regression model $Y_i$ is a the sum of the overall mean $\beta _0$ and the sampling error $\epsilon _i$

2 $$\begin{aligned} Y_i = X_0 \beta _0 + \epsilon _{i} \equiv \beta _0 + \epsilon _i\ \text {for i} = 1, \ldots ,I \end{aligned}$$

$\epsilon _i$ represents the $i^{th}$ deviation from the overall mean. The deviations are assumed to be identical, independent, centrally located around zero and with constant variance

3 $$\begin{aligned} mean(\epsilon _i )&= 0 \nonumber \\ var(\epsilon _i)&= \sigma ^2 \nonumber \\&\text {implying therefore that} \nonumber \\ mean(Y_i)&= \beta _0 \nonumber \\ var(Y_i )&= \sigma ^2 \nonumber \\ cov(Y_i,Y_i')&= 0\;\text {for any}\ i \ne i'. \end{aligned}$$

### Estimating $\beta _0$ and var($\hat{\beta }_0$)

Based on the principle of OLS, simple algebra yields

4 $$\begin{aligned} \hat{\beta }_0&= \bar{Y} \nonumber \\&= \frac{\sum _{i=1}^{I} Y_i}{I} \nonumber \\ \hat{\sigma }^2&= \frac{\sum _{i=1}^{I} (Y_i - \hat{\beta }_0)^2}{I-1} \nonumber \\ var(\hat{\beta }_0)&= \frac{\hat{\sigma }^2}{I} \end{aligned}$$

These estimates are valid irrespective of the actual distribution of *Y* (or of $\epsilon$).

### Inference about $\beta _0$

To compute the confidence intervals (CI) for $\hat{\beta }_0$ and perform hypothesis tests about $\beta _0$, it is essential to know its sampling distribution. Essentially, we want to know, if we take another sample of *Y* and compute another value of $\hat{\beta }_0$, how close it will be to the original estimate. Once we know the distribution, we can identify its lower and upper critical values, and the rejection region at $\alpha$(typically $5\%$) level of significance. Resampling methods e.g. bootstrap, generate the sampling distribution by permuting (e.g. sampling *I* times with replacement) *Y* many times, each time re-calculating $\hat{\beta }_0$. This method is computationally expensive, especially in complex models. Conventionally, a known distribution is assumed. Since $\hat{\beta }_0$ is a function of $Y_i$ (see equations 4), and $Y_i$ is function of $\epsilon _i$ (see equation 2), if the sampling distribution of $\epsilon _i$ is known, the sampling distributions of $Y_i$ and $\hat{\beta }_0$ is automatically known. The normal distribution is the standard assumption because it simplifies the calculation and inference.

5 $$\begin{aligned} \epsilon _i&\sim N(0, \sigma ^2)\;\text {implying} \nonumber \\ Y_i&\sim N(\beta _0, \sigma ^2)\;\text {so that} \nonumber \\ \hat{\beta }_0&\sim N(\beta _0, \frac{\sigma ^2}{I}) \end{aligned}$$

Consequently, the OLS estimates in equations (4) are also ML estimates.

When $\sigma ^2$ is known or the sample size *I* is sufficiently large, the Wald CIs and Wald test statistics can be used for inference. Often, the sample size is small ($I < 30$) and $\sigma ^2$ unknown, and proceeding with inference based on the asymptotic normality of $\hat{\beta }_0$ would be misleading. In such cases, the actual coverage probability of the Wald CIs often falls below the nominal confidence coefficient. By replacing $\sigma ^2$ in $\hat{\beta }_0 \sim N(\beta _0, \frac{\sigma ^2}{I})$ with its estimate $\hat{\sigma }^2$ from equation (4), elementary probability theory implies that the exact distribution of $\hat{\beta }_0$ is the *t*-distribution with $I-1$ degrees of freedom. When there are *C* covariates in the regression model, the *t*-distribution will have $I- C - 1$ degrees of freedom. Like the normal distribution, the *t*-distribution is symmetric and bell-shaped but with heavier tails. For large sample sizes, the two distributions are practically the same.

## Connecting meta-analysis to the linear model

Consider the randomized complete block design in the analysis of variance where subjects are grouped into *J* homogeneous populations (the blocks), and treatment is assigned randomly to each subject within the blocks. Let $Y_{ij}$ and $X_{ij}$ denote the outcome response and the variable of interest (a treatment/intervention) from subject *i* in population *j*, respectively. Other blocking variables $Z_j = (Z_{j0}, \ldots , Z_{jC})$ can be utilized to further reduce the variation between the subjects within a block.

Similar to such a design, meta-analysis is a study of separate studies addressing the same research question and with a similar design to integrate the study results. In practice, obtaining the individual patient data $Y_{ij}$ or sufficient summaries e.g. $\sum {Y_{ij}} = Y_j$ is time-consuming, expensive and often impossible. Conventionally, a generic model is directly fitted to the study parameter estimates $\hat{\beta }_j$ because it simplifies the analysis. In the following sections, we review the main models used in this context: the formulation, assumptions, estimation, inference and the effects of violation of assumptions on estimation and inference.

### The CE linear regression model

Let $Z_0 = 1$. Similar to equation (2), a study parameter estimate $\hat{\beta }_j$ is the sum of an average value $\mu _0$ and the study’s sampling error $\xi _j$

6 $$\begin{aligned} \hat{\beta }_j = Z_0\mu _0 + \xi _j \equiv \mu _0 + \xi _j ~\text {for j} = 1, \ldots , J \end{aligned}$$

#### Estimating $\mu _0$ and var($\hat{\mu }_0$)

The error terms $\xi _j$ are assumed to be independent and centrally located around zero i.e. $E(\xi _j) = 0$. However, unlike in equation (3), their variances $var(\xi _{j}) = \nu _j^2$ are variable implying that the parameter estimates do not have the same reliability. This feature is equivalent to heteroscedasticity in the classical linear regression model (2). To account for the differences in reliability, the estimation equations (4) are modified by assigning a weight to each data point. Conventionally, the weights given are inverse to the within-study variance $w_j = \frac{1}{\nu _j^2}$ so that precise and/or larger studies with smaller variances (more reliable information) get more weight. This is the inverse-variance (IV) weighting scheme.

Based on the principle of WLS, the modified estimation equations are

7 $$\begin{aligned} \hat{\mu }_0&= \bar{\beta }_{ce} = \frac{\sum _{j=1}^{J}w_j \hat{\beta }_j}{\sum _{j=1}^{J}w_j}\ \text {and} \nonumber \\ var(\bar{\beta }_{ce})&= \sum _{j=1}^{J}w_j \equiv \sum _{j=1}^{J}\frac{1}{\nu ^2_j} \end{aligned}$$

Like OLS, WLS does not require knowledge of the distribution of the study parameter estimates $\hat{\beta }_j$.

#### Inference about $\bar{\beta }_{ce}$

To compute the CIs for the average of the study parameter estimates $\bar{\beta }_{ce}$ and conduct hypothesis tests about it, we need to know its sampling distribution (or equivalently of $\hat{\beta }_j$ or $\xi _j$). Analogous to the distribution specifications (5), the standard assumption is the normal distribution $\xi _j \sim N(0, \nu _j^2)$ so that

8 $$\begin{aligned} \hat{\beta }_j&\sim N(\mu _0, \nu _j^2)\ \text {and consequently} \nonumber \\ \bar{\beta }_{ce}&\sim N\bigg (\mu _0, \sum _{j=1}^{J}\frac{1}{\nu ^2_j}\bigg ) \end{aligned}$$

The within-study variance $\nu _j^2$ is a random variable. Ideally, a variance function should be estimated by regressing the squared residuals or the sample variances against an “appropriate” predictor variable. The fitted values from the variance function are then used to obtain $\nu _j^2$ [19]. Unfortunately, information on the “appropriate” predictor variable is never available. Conventionally, $\nu _j^2$ is replaced with the estimated study sample variances $\hat{\nu }_j^2$ so that

9 $$\begin{aligned} \hat{\beta }_j&\sim N(\mu _0, \hat{\nu }_j^2)\ \text {and consequently} \nonumber \\ \bar{\beta }_{ce}&\sim t_{J-1}\bigg (\mu _0, \sum _{j=1}^{J}\frac{1}{\hat{\nu }^2_j}\bigg ) \end{aligned}$$

The inference is now approximate because the estimation of $\hat{\nu }_j^2$ introduces another source of uncertainty. The approximate Wald CIs are known to perform poorly but their use is common. The bootstrap CIs are more conservative than the Wald CIs [20] but their use is seldom. The direct use of $\hat{\nu }_j^2$ leads to underestimation of $\sum _{j=1}^{J}w_j$. Consequently, the CIs will be narrower and the *p*-values smaller than when the uncertainty would be accounted for. When the number of studies in the meta-analysis is large enough, the direct use of sample variances to estimate the unknown within-study variances may be justified. This is because the weights become essentially irrelevant. Alternative weighting schemes use a function of the study size only. Some of the arguments for not using the within-study variance are

- To avoid giving large weights to small but precise studies especially when there are few studies.
- To avoid the estimation error in the within-study variance [21].
- To assign uniform weight regardless of the metric of the effect size [22].

### Overdispersion

There is overdispersion when the observed variation in the data is more than explained by a model. Ignoring the excess variation underestimates the standard errors of the regression coefficients resulting in misleading inference. From the goodness of fit perspective, over-dispersion indicates a lack of fit. The inadequacy in the model maybe due to the omission of important study characteristics in the model, data coming from a population having different sub-populations, or the presence of correlation [6, 23]. When there are sufficient number of studies in the meta-analysis, some of the excess variation can be attributed to known study characteristics in a meta-regression. However, this is not common practice because many meta-analyses do not have a sufficient number of studies to incorporate study characteristics into the model. Even when there are adequate studies and there are known study characteristics, the CE model is used because the WLS computations involved in a meta-regression become complex or inapplicable.

We briefly review the current techniques to handle overdispersion in the CE model (6). These techniques treat overdispersion as a nuisance and handle its impact by inflating the study variances. If overdispersion is due to the CE model overlooking differences among $\hat{\beta }$ that may be important to recognize, making an adjustment for overdispersion will not address the inadequacy.

### Likelihood approaches to handle overdispersion

#### The RE linear regression model

A study parameter $\delta _j$ is added to the regression equation 6:

10 $$\begin{aligned} \hat{\beta }_j = \mu _0 + \delta _j + \xi _j \end{aligned}$$

Conventionally, the $\delta _j$ is assumed to be normally distributed

11 $$\begin{aligned} \delta _j \sim N(0, \tau ^2) \end{aligned}$$

Rewriting equations (10) and (11) as

12 $$\begin{aligned} \hat{\beta }_j&= \mu _j + \xi _j \nonumber \\ \mu _j&\sim N(\mu _0, \tau ^2) \end{aligned}$$

implies that the expected study means $(\mu _1, \ldots , \mu _J)$ are normal random variables from a population of studies with mean $\mu _0$ and variance $\tau ^2$. $\tau ^2$ represents the variability between the study means. The two random components $\xi _j$ and $\delta _j$ in equation (10) are uncorrelated. It is automatic then that

13 $$\begin{aligned} var(\hat{\beta }_j)&= \hat{\nu }_j^2 + \tau ^2 \nonumber \\ \hat{\beta }_j&\sim N(\mu _0, \hat{\nu }_j^2 + \tau ^2). \end{aligned}$$

There are different methods to obtain an estimate of $\tau ^2$ including the method of moments (MOM), ML and restricted maximum likelihood (REML) [24]. The accuracy of the estimated $\tau ^2$ depends on the estimation method and the number of studies *I*. The REML estimator is more efficient and reliable than the popular Dersimonian-Liard MOM estimator [25] even when there are few studies ($J\le 5$) [26]. Once $\tau ^2$ is estimated, the weights in equations (7) are replaced with $w^*_j = \frac{1}{(\hat{\nu }_j^2 + \hat{\tau }^2)}$. The modified estimation equations are

14 $$\begin{aligned} \bar{\beta }_{re}&= \frac{\sum _{j=1}^{J}w^*_j \hat{\beta }_j}{\sum _{j=1}^{J}w^*_j}\ \text {and} \nonumber \\ var(\bar{\beta }_{re})&= \sum _{j=1}^{J}w^*_j = \sum _{j=1}^{J}\frac{1}{\hat{\nu }^2_j + \hat{\tau }^2}. \end{aligned}$$

The addition of $\hat{\tau }^2$ to the study variances increases the standard errors of the weighted mean by penalizing studies with small variance (usually the large studies). As $\tau ^2$ increases, the distribution of the weights between the studies become increasingly even. This distortion of weights may lead to contradictory conclusion [1, 5].

#### Thompson and Sharp [27] multiplicative model 

A multiplicative parameter is added to the CE model (6) to expand the study variances by a constant $\phi$

15 $$\begin{aligned} \hat{\beta }_j&= \mu _0 + \xi _j\sqrt{\phi }\ \text{ which } \text{ implies } \nonumber \\ \hat{\beta }_j&\sim N(\mu _0, \phi \hat{\nu }_j^2) \end{aligned}$$

$\phi$ represents the degree of overdispersion. It is estimated as the mean square error from regressing $\hat{\beta} _j$ against a constant with weights $w_j = \frac{1}{\hat{\nu }^2}$ and extracting the mean squared error. The estimate is set to 1 if its < 1. After estimating $\phi$, the new weights $w_j^* = \frac{1}{\hat{\phi }\hat{\nu }^2}$ are plugged into equations (14). In the equation for $\hat{\mu _0}$, $\hat{\phi }$ falls off implying that the estimated average value $\bar{\beta }_{mts}$ will be identical to $\bar{\beta }_{ce}$ and the standard error will be inflated by a factor $\sqrt{\phi }$.

There barely are significant differences in terms of Akaike Information Criterion (AIC) between this model and the RE model (13) with the ML $\tau ^2$ estimator [28]. Despite its simplicity, its use is discouraged - the rationale for using a multiplicative factor for variance inflation is weak [27, 29].

#### Kulinskaya and Olkin [30] multiplicative model

The $\phi$ in model (15) is replaced by a linear function of the study sizes $N_j$ and the intra-cluster correlation (ICC) $\rho$ thus allowing for the deflation of within-study variance as well.

16 $$\begin{aligned} \hat{\beta }_j&\sim N\bigg (\mu _0, \frac{(1 - \rho )(1 + N_j \gamma )}{N_j} \hat{\nu }_j^2\bigg )\ \text {for} \nonumber \\ \gamma&= \frac{\rho }{1 - \rho } > \frac{-1}{max(N_j)} \end{aligned}$$

There are a variety of methods to estimate the parameter $\gamma$ including MOM, ML, REML, Mandel-Paule none of which is uniformly the best, regardless of the criterion [30]. After obtaining an estimate of $\gamma$, the new weights are plugged in equations (14) to obtain $\bar{\beta }_{ko}$ and its variance. This model is rarely applied possibly because

- The coverage of the resulting approximate Wald CI is considerably lower than nominal compared to the RE model.
- Underdispersion is less frequent in practice.
- Allowing for deflation of variance is discouraged [28].

#### Approximate inference about $\hat{\mu }_0$ in the RE and the multiplicative models

Because $\hat{\beta }_j$ is assumed to be a normal variate, the approximate sampling distribution of $\hat{\mu }_0$ is often assumed to be normal. However, the approximate Wald test and approximate Wald CI have a downward bias. This should be resolved by using approximate *t*-distribution with $J-1$ degrees of freedom [15, 31, 32].

### Quasi-likelihood approaches to handle overdispersion

The idea is to modify the estimation equations from a corresponding likelihood method to make them sufficiently flexible and “work” at the same time. The new estimation equations may not correspond to a known or any distribution hence the distribution of $\hat{\beta }_j$ is considered unspecified.

#### IVhet model [1]

New estimation equations are derived based on concepts from the multiplicative models (15) and (16) and the RE model (13).

The within-study variances in the estimations equations (7) are expanded by an overdispersion parameter $\psi _j$; a function of ICC $\rho _j$ which is a function of the Dersimonian-Liard MOM estimator $\tau ^2$ from the RE model (13) yielding

17 $$\begin{aligned} w_j^*&= \frac{1}{\psi _j \hat{\nu }_j^2}\ \text {where} \nonumber \\ \psi _j&= \frac{1}{1 -\rho _j}\ \text {where} \nonumber \\ \rho _j&= \frac{\hat{\tau }^2}{\hat{\tau }^2 + \hat{\nu }_j^2} \end{aligned}$$

$\psi _j$ falls off in the computation of $\hat{\mu }_0$ implying that the estimated value $\bar{\beta }_{ivhet}$ will be identical to the $\bar{\beta }_{ce}$ and $\bar{\beta }_{mts}$. Its variance is inflated to $\sum _{j=1}^{J}\frac{1}{\psi _j \hat{\nu }_j^2}$.

Doi et al. [1] compared the coverage probability of the approximate Wald CI of $\bar{\beta }_{ivhet}$ from this model an﻿d $\bar{\beta }_{re}$ from the RE model (13) with Dersimonian-Liard MOM $\tau ^2$ estimator; the former had a coverage probability close to the nominal level.

#### QE model [14]

New estimation equations using synthetic weights that incorporate study quality are derived. The synthetic weights - the reciprocal of the sum of the estimated within-study variances and an internal bias $\phi _j$ - are created through a quality score $Q_j$:

18 $$\begin{aligned} w^*_j&= \frac{1}{(\hat{\nu }_j^2 + \hat{\phi }^2)} \approx \frac{Q_j}{\hat{\nu }_j^2} + \hat{\gamma }_j \nonumber \\ \hat{\mu }_0&= \bar{\beta }_{qe} = \frac{\sum _{j=1}^{J}w^*_j \hat{\beta }_j}{\sum _{j=1}^{J}w^*_j} \nonumber \\ var(\bar{\beta }_{qe})&= \sum _{j=1}^{J}[(w^*_j)^2(\hat{\nu }_j^2 + \hat{\tau }_j)] \end{aligned}$$

where $\hat{\gamma }_j$ is an adjustment [14] (function of $Q_j$ and $\hat{\nu }_j^2$) to prevent the possibility of negative synthetic weights. $\tau ^2$ is the Dersimonian-Liard MOM estimator from the RE model (13). When quality information is available, Doi et al. [14] showed that the QE model handles the effect of overdispersion better than the RE model with the Dersimonian-Liard MOM $\tau ^2$ estimator.

The attractiveness of the quasi-likelihood approach is that it requires fewer assumptions than a full likelihood approach but the number of studies in the meta-analysis should be sufficiently large for asymptotic inference. The main disadvantage is that model comparison procedures using the Likelihood Ratio (LR) tests, AIC or Bayesian Information Criteria (BIC) are not possible because the distribution of $\hat{\beta }$ is not specified [33].

## Meta-analysis of proportions in the framework of linear models

We refer to the study *j* with a fixed number of binary responses $Y_{ij}$ generically labelled “success” (alive/healthy/cured) and “failure” (dead/sick/not cured). Let $n_j$ be the number of “successes” and $N_j$ be the sum of ‘successes’ and ‘failures’. It is natural to assume that $n_j$ is a binomially distributed random variable with parameters $N_j$ and $\pi _j$; the probability of “success”. The distribution is denoted by

19 $$\begin{aligned} n_j \sim bin(\pi _j, N_j) \end{aligned}$$

The ${\pi _j}s$ are the parameters of interest. The MOM and the ML estimator for $\pi _j$ is $\hat{\pi }_j = \frac{n_j}{N_j}$. Its variance is

20 $$\begin{aligned} \hat{\nu }_j^2 = \frac{\hat{\pi }_j (1 - \hat{\pi }_j)}{N_j} = \frac{n_j (N_j - n_j)}{N_j^3} \end{aligned}$$

### Problems in one group meta-analysis

Let $\hat{w}_j^{-1} = \hat{\nu }_j^2$, the WLS and the ML estimate for the average value $\bar{\pi }_{iv}$ is

21 $$\begin{aligned} \bar{\pi }_{iv}= & {} \frac{\sum _{j=1}^{J} \hat{w}_j \frac{n_j}{N_j}}{\sum _{j=1}^{J} \hat{w}_j} \nonumber \\= & {} \frac{\sum _{j=1}^{J} \frac{N_j^2}{N_j - n_j}}{\sum _{j=1}^{J} \frac{N_j^3}{n_j * (N_j - n_j)}} \end{aligned}$$

1. When $n_j=0$ or $n_j= N_j$, $\hat{\nu }_j^2=0$ implying $\hat{w}_j$ is undefined leading to the exclusion of the study from the analysis. To keep the study, an ad hoc continuity correction is applied. The exclusion of studies and application of the continuity correction can result in biased estimates [34].
2. When *J* is small and/or $\hat{\pi }_j$ is near 0/1, the distribution of $\hat{\pi }_j$ is likely to be skewed and discrete. The symmetricity of the normal (or *t*) distribution allocates equal probability to each tail. This is reasonable whenever the proportions are all around 0.5. However, when $\hat{\pi }$ is near zero or one, the symmetricity violates the natural boundaries of the $\pi$. It is then possible to have studies with confidence intervals outside the admissible interval [0, 1] in the forest plot.
3. The within-study variance $\hat{\nu }_j^2$ is a function of mean $\hat{\pi }_j$. This mean-variance relationship has two implications:
  - Ignoring the dependence may bias the estimates for $\bar{\pi }_{iv}$ and its variance [35, 36].
  - The domains of $\hat{\pi }_j$ and $\hat{\nu }_j^2$ are constrained (in contrast, the variances in the normal distribution are unbound and independent of the mean). As $\hat{\pi }_j$ moves towards 0 or 1, $\hat{\nu }_j^2$ moves towards 0, is highest when $\hat{\pi }_j = 0.5$, and never exceeds $\frac{0.25}{N_j}$. This then constrains the domain of the dispersion parameters. Thus, correcting for overdispersion without formal modeling (i.e. support from the data) may be misleading.

Several actions are taken in an attempt to achieve symmetricity and stabilize the study variances. Transformations such as logit and arcsine-based have been the obvious recourse for the longest time simply due to their mathematical simplicity. The Stata package metaprop popularized the Freeman-Tukey double arcsine transformation (FTT) [37]. Following the high recommendation of the FTT by Doi et al. [38] the Stata procedures metaan and metan have also implemented the FTT. However, its conversion formula has a structural defect. The back-transformed values can sometimes fall outside the admissible [0, 1] range depending on the “overall sample size”. There is no consensus nor justification for what should be the “overall study size”; the harmonic, geometric, or arithmetic mean of the study sizes. Doi et al. [38] discourages using any of the means and recommends the inverse variance [39] as the “overall sample size” which has the benefit of avoiding the inadmissible values. This structural defect exists simply because the FTT was never intended for use in meta-analysis but for inference in a single study [37]. Moreover, the transformation obscures the true nature of the data by breaking the mean-variance relationship in binomial data in order to stabilize the variance. More inadequacies of the Freeman-Tukey double arcsine transformation are detailed elsewhere [39–43]. Theoretical derivations and simulations have demonstrated considerable biases in the parameter estimates arising from the logit and the arcsine transformations [44, 45].

### Problems in two group meta-analysis

In meta-analysis comparing two proportions, the log-transformed estimated relative risks or odds ratios and their standard errors are used e.g. in metan, metaan, mvmeta and meta. When there are no events in either group, the estimated ratio (RR or OR) is undefined or 0, and such studies are excluded from the analysis. It is argued that they provide no information on which group has the higher risk when using the OR as an outcome measure. However, simulations have shown that these studies contain information and can influence the conclusion of the meta-analysis [46]. To avoid excluding these studies, an ad hoc continuity correction is applied. It is possible that the addition of a continuity correction could swamp the data and have a marked effect on the results. If there are more than two groups, meta offers support for meta-regression. The common practice however is to examine the differences informally in a subgroup analysis. This ignores the covariance among variables which can lead to spurious significant effects, confounded effects [47], and invalid standard errors. A simulation study [48] showed that the inverse-variance weights methodology was by far the most unreliable in meta-analysis comparing two proportions.

### The problem of data reduction

Corresponding to each study, there is a probability distribution of the binomial variables. In order to use the point estimates of the binomial parameters and their estimated variances, we perform data reduction and certainly lose some information about the original data. In presence of overdispersion, the addition of $\hat{\tau }^2$ to the study variances to compute the RE weights introduces a distortion to the data. The “new RE” data share little in common with the original data and therefore there is no guarantee that the corresponding solution is valid for the original inference problem. The motivation for the data reduction is minimizing the computational effort required to solve the problem e.g. use of the MOM $\hat{\tau }^2$ in WLS. The principle of ML is a data reduction method that does not discard important information about the unknown parameters [49]. However, if data reduction is done prior to application of ML, the discarded data will never be analyzed.

Given these limitations, it is best to abandon the procedures based on approximation to the normal distribution and use/develop a better modeling approach that 1. is more appropriate for the natural distribution of the proportions, and 2. does not discard important information about the unknown parameters.

### Meta-analysis of binomial proportions in the framework of the GLM and GLMM

#### Choice between models

To model proportions, the binomial distribution is generally appropriate or very close to appropriate. In a binomial model, overdispersion occurs when the mean-variance relationship breaks down due to the non-constancy of the binomial parameter among the studies. As a consequence, the variation in the observed proportions will exceed the variance of the binomial distribution. The systematic differences among the studies can be incorporated in a hierarchical model using an indicator for each study. These indicators are assumed to follow a normal or beta distribution.

The pool of individuals in the studies is expected to differ by important factors such as intervention assignment, study protocol, clinical setting, etc. This information is easily incorporated into a hierarchical model in a meta-regression as covariates. When there are covariates, the appropriateness of a regression model and the parsimony of the model fit must always be considered. Significant covariates could be dropped due to low statistical power [47] if there are too many covariates in the model. In comparative analysis, the covariate of interest might not be statistically significant. Nonetheless, because it is central to the purpose of the meta-analysis, it is sensible to keep it in the model. It may also reduce bias in the estimated effects of the other covariates. To assess the parsimony of the model fit, both the Wald test and the LR tests can be conducted in hypothesis testing. Should there be a conflict between the Wald and the LR test, the LR test result is a more powerful test. The results of these tests provide evidence on whether the model fits the data reasonably well. Non-convergence and inestimable effects are other indications of lack of fit for which we recommend fitting a simpler model. Besides the Wald and the LR tests, one can also use the BIC to select the optimal model with the lowest value.

#### Computing

We fit the models presented in the following sections using the Stata package metapreg. With a connection to the internet, directly install the program into Stata

. ssc install metapreg

After the installation is complete, you can open the help window for a detailed description of the command options and demonstration examples. The datasets used in the demonstration examples are available with a click.

. help metapreg

Table 1 highlights some features of metapreg. By default, the logistic regression model with varying intercept-only is fitted. Whenever there are fewer than three studies in an analysis, the procedure automatically switches to the ordinary logistic regression model with a constant intercept only. When there are zero counts in the data, no ad-hoc continuity correction or data imputation is required. In meta-regression, both categorical (string variables) and continuous study-level covariates are permitted. There is no limit on the number of covariates allowed but only the interaction between the first covariate and the rest is permitted; for simplicity. The coefficients from the logistic regression models can be challenging to interpret due to the nonlinearity of the logistic function. The model coefficients are further processed by employing marginal standardization [50–52] and simulations of the posterior distribution to yield meaningful results.

**Table 1:** Some of the options in metapreg

Option in metapreg	Descriptions	Remarks
model()	Specifies the type of the model.	There are three types of the model; model(hexact), model(random) which is also design(mixed), and model(fixed).
design()	Specifies the structure of data and the covariance structure of the random effects.	There are five different structures of data anticipated; design(general), design(comparative), design(pcbnetwork), design(mcbnetwork) and design(abnetwork). When there are two random effects in a comparative analysis, the possible covariance structures are design(comparative, cov(independent)), design(comparative, cov(unstructured))
smooth()	Requests for the model-based study estimates.	Provides a visual assessment of whether the model is a good data summary.
cimethod()	Specifies the type of confidence intervals for the study-specific estimates as displayed in the forest plot.	For proportions, the possibilities are cimethod(exact), cimethod(wald), cimethod(wilson), cimethod(agresti), and cimethod(jeffreys). For comparative and paired data, only cimethod(cml) is allowed when computing the CI for the probability ratios. For matched data, the only possibility for probability ratios is cimethod(koopman). For the odds ratios, the possibilities are cimethod(exact), cimethod(cornfield) and cimethod(woolf).
nomc	Instructs the program not to conduct model comparison in meta-regression.	Not fitting simpler models than what is requested saves time.
by()	Requests the summary estimates at the unique values of the by variable.	Can be useful when the model contains multiple covariates and grouped summary estimates are required.
stratify	Together with the option by(), the stratify option makes it possible to fit separate models in each group of data in the by variable, but present the results in one table and forest plot.	In contrast; the prefix command by: would print out separate tables and graphs for each sub-analysis.
sumtable()	Indicates the type of summary estimates to display.	The possibilities are sumtable(logit), sumtable(abs). When there are categorical data in the model, sumtable(rr) or sumtable(or) are also allowed. All tables are displayed with sumtable(all). By default, none of the tables are displayed.
outplot()	specifies which statistics to display on the forest plot.	outplot(abs) displays the study-specific and summary proportions. outplot(rr) and outplot(or) displays the study-specific probability and odds ratios when data are from comparative, paired, or matched studies. In the case of network meta-analysis, outplot(rr) and outplot(or) displays the summary probability and odds ratio(s) of the multiple treatments relative to the specified reference.

metapreg can also perform stratified analysis and model comparisons; by leaving out the interaction terms or one covariate at a time. When there are no covariates in the logistic regression model, the $I^2$ statistic by Zhou & Dendukuri [53] is also computed and presented alongside the $\hat{\tau }^2$. The statistic accounts appropriately for the variability of the within-study variance and the mean-variance relationship across studies.

To ease exploratory analysis, interpretation and communication of the results from the meta-analysis are presented in a forest plot. When presenting the observed proportions, the Wald, Wilson, Agresti-Coull, Jeffreys, or Clopper-Pearson CIs can be computed. In a comparative meta-analysis, a forest plot of the study-specific RR or OR can be requested. In an arm-based network meta-analysis, the forest plot presents the summary proportions relative to a “reference group”. The “reference group” is one of the groups; automatically assigned by the procedure or explicitly specified by the meta-analyst.

The code to reproduce the analyses in the next sections can be downloaded at https://github.com/VNyaga/Metapreg/blob/master/metapreg-article-code.do.

#### One group meta-analysis

##### Logistic regression model with constant intercept-only - CE model

Assuming that the proportion of events in all the studies is the same treats the studies as identical replications of each other and implies that each $\pi _j = \pi$. It follows then that

22 $$\begin{aligned} n_j \sim bin(\pi , N_j) \end{aligned}$$

The ML estimator $\hat{\pi }_c$ for $\pi$ is

23 $$\begin{aligned} \hat{\pi }_{c} = \frac{\sum _{j=1}^{J}n_j}{\sum _{j=1}^{J}N_j} \end{aligned}$$

The derivation of $\hat{\pi }$ is shown in the additional supporting information. A similar estimate is obtained when working with the constant intercept-only logistic regression model where the binomial parameter is expressed as $\pi = \frac{e^{\beta _0}}{1-e^{\beta _0}}$.

##### Retrieving the study weights

A relation exists between WLS and ML estimation of the logistic regression model parameters which uses the Newton-Raphson algorithm. The algorithm repeatedly use WLS, with weights changing at each iteration. Each step involves a normal approximation to the log-likelihood based on the current solution to find an updated solution by WLS. After convergence, the solution barely changes in successive iterations.

The maximized log-likelihood $L(\hat{\pi })$ contains all current information about the $\pi$ from all the studies. The relative “value” of the information provided by a study is encapsulated in its contribution $L_j(\hat{\pi })$ to the maximized log-likelihood as

24 $$\begin{aligned} L_j(\hat{\pi }_{c}) \propto n_j*log \bigg (\hat{\pi }_{c} \bigg ) + (N_j - n_j)*log \bigg (1 - \hat{\pi }_{c} \bigg ) \end{aligned}$$

Let $\hat{\eta } = log \bigg (\frac{\hat{\pi }_{c}}{1 - \hat{\pi }_{c}} \bigg )$. The information $L_j(\hat{\pi })$ is approximately equivalent to normally distributed ‘working’ dependent variable $z_j$ with mean $\hat{\eta }$ and variance $\nu ^{2}_j$ [54]

25 $$\begin{aligned} z_j&= \hat{\eta } + \frac{(1 + e^{\hat{\eta }})^2}{e^{\hat{\eta }}} \bigg (\frac{n_j}{N_j} - \frac{e^{\hat{\eta }}}{1 + e^{\hat{\eta }}} \bigg ) \nonumber \\ \nu ^{2}_j&= \frac{(1 + e^{\hat{\eta }})^2}{N_j \times e^{\hat{\eta }}} \nonumber \\&= \frac{1}{N_j \hat{\pi }_{c} (1 - \hat{\pi }_{c})} \end{aligned}$$

The weights that sum to 1 are then computed as

26 $$\begin{aligned} w^c_j = \frac{\frac{1}{\nu ^{2}_j}}{\sum _{j=1}^{J}\frac{1}{\nu ^{2}_j}} \equiv \frac{N_j}{\sum _{j=1}^{J} N_j} \end{aligned}$$

##### Inference about the population mean

To compute the Wald CI for $\hat{\pi }_{c}$, its asymptotic variance (see derivation in the additional supporting information) is

27 $$\begin{aligned} var(\hat{\pi }_{c}) = \frac{\hat{\pi }_{c}(1 - \hat{\pi }_{c})}{\sum _{j=1}^{J}N_j} \end{aligned}$$

When $\hat{\pi }_{c}$ is zero or one, the asymptotic variance is 0 and the Wald CI degenerates. Since $\pi$ is assumed to be the same in each study, the sum of all “successes” is again a binomial variable. Therefore

28 $$\begin{aligned} \sum _{j=1}^{J}n_j \sim bin (\pi , \sum _{j=1}^{J}N_j) \end{aligned}$$

The Clopper-Pearson $1 - \alpha$ CI for $\hat{\pi }_{c}$ is [L, U] with L and U as the solution to the equations [55]

29 $$\begin{aligned} p(x \ge \sum _{j=1}^{J}n_j)&= \frac{\alpha }{2}\ \text {and} \nonumber \\ p(x \le \sum _{j=1}^{J}n_j)&= \frac{\alpha }{2}\ \text {for}\;x= 0, \ldots \sum _{j=1}^{J}N_j \end{aligned}$$

The coverage of the Clopper-Pearson CI is at least $1 - \alpha$. The coverage probabilities of the score CI tend to be near the nominal value except for some values of $\pi$ very close to zero or one [56]. The score CI is computed as

30 $$\begin{aligned} \frac{\hat{\pi }_{c} + \frac{z}{2\sum _{j=1}^{J}N_j} \pm z \sqrt{\hat{\pi }_{c}(1 - \hat{\pi }_{c}) + \frac{4\sum _{j=1}^{J}N_j}{\sum _{j=1}^{J}N_j} } }{1 + \frac{z}{\sum _{j=1}^{J}N_j}} \end{aligned}$$

where *z* is the $\frac{\alpha }{2}$ percentile of the standard normal distribution.

##### Example I - A systematic review on the efficacy of cold coagulation as a treatment for cervical intraepithelial neoplasia.

In the review, Dolman et al. [57] fitted the IV RE model with metan. The estimate of heterogeneity was taken from the Mantel-Haenszel model. We have previously used this dataset to demonstrate metaprop and metaprop_one in Nyaga, Arbyn and Aerts [58]. Here, we use the dataset to delineate the conceptual differences between the constant and varying intercept-only logistic regression models.


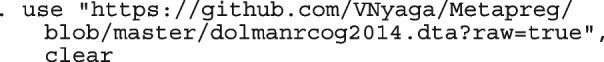


The following code performs the meta-analysis using the constant intercept-only logistic regression model which is equivalent to using the binomial distribution. 
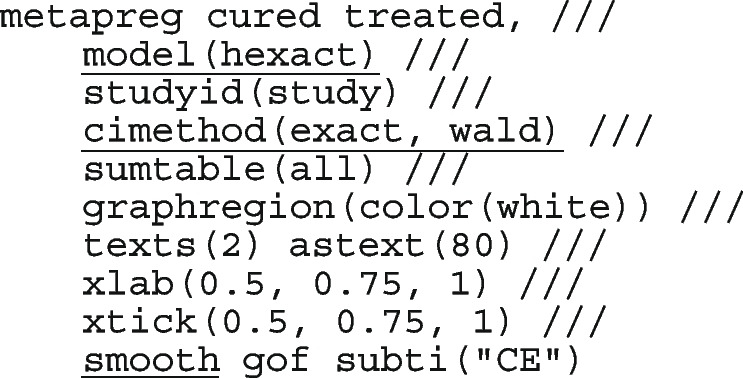


The option model(hexact) requests for the estimate of the mean from the exact binomial distribution. cimethod(exact, wald) requests the 95% Clopper-Pearson (exact) CI for the observed proportions and the Wald CI for the population mean, respectively. smooth requests for the expected/fitted/smoothed/model-based proportions and their corresponding 95% Wald CI. gof requests for the model’s AIC and BIC. The model structure fitted to the data is 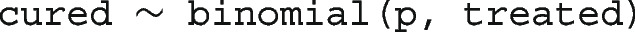


Several tables are displayed in the results window but are not presented here. They contain information on the goodness-of-fit criterion, the study-specific and population-averaged inferences.

**Fig. 1:**
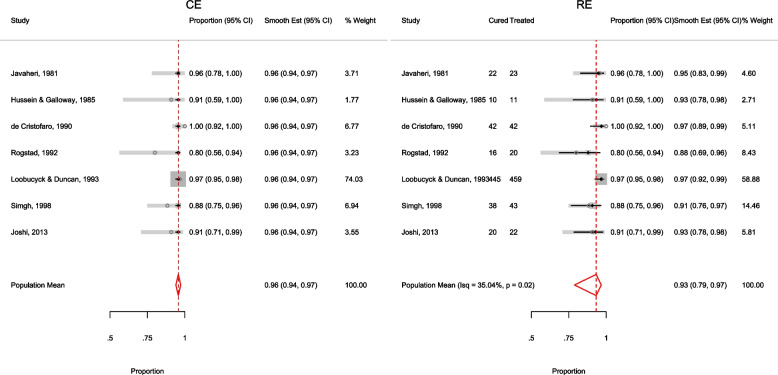
Forest plot from a meta-analysis on the efficacy of cold coagulation using the (CE) binomial and the RE logistic regression. Confidence intervals for individual studies are computed using the Clopper-Pearson's method

The forest plot from the meta-analysis is presented in the left graph in Fig. 1. The gray circles and lines represent the observed proportions and their 95% CI. The black dots and lines represent the fitted proportions and their 95% CI. The red diamond represents the population mean and its 95% CI. The dark gray boxes represent the study weight. The model implies that the proportion of patients cured is the same in each study, hence, the model-based estimates (black dots and lines) are the same in the seven studies. However, the 95% CI of the observed 0.80 [0.56, 0.94] versus the expected 0.96[0.94, 0.97] proportion in the Rogstad, 1992 study barely overlap suggesting that the model fits the data point poorly. Furthermore, the non-overlapping 95% CI’s of the observed proportions in the largest study Loobucyck & Duncan, 1993 0.97 [0.95, 0.98] and the Rogstad, 1992 study 0.80 [0.56, 0.94] suggests that the proportion of patients cured might not all be the same between the seven studies.

##### Logistic regression model with varying intercept-only - RE model

We reformulate the distribution as

31 $$\begin{aligned} n_j \sim bin(\pi _j, N_j) \end{aligned}$$

Using the beta distribution to model the variation of $\pi$ among the studies is ideal because it describes the distribution of a continuous variable in the interval [0, 1]

32 $$\begin{aligned} \pi _j \sim beta(a, b)\ \text {for}\ a, b > 0 \end{aligned}$$

To ensure unimodality of the random-effect distribution and hence the identifiability of $\pi$, *a* and *b* must be $\ge$ 1. The beta distribution is naturally conjugate to the binomial distribution. This greatly simplifies the computations in estimating the model parameter estimates and their interpretation since

33 $$\begin{aligned} E(\pi _j)&= \frac{a}{a + b} \nonumber \\ var(\pi _j)&= \frac{ab}{(a + b)^2(1 + a + b)} \end{aligned}$$

However, fitting the beta-binomial model outside the Bayesian setting is complex and requires extensive programming. The user-written Stata command betabin fits binomial regression models allowing for beta overdispersion.

It is computationally convenient to employ the logit function on the binomial parameter $\pi _j$ and add a parameter $\delta _j$ representing the unmeasured or omitted study characteristics responsible for the variation of $\pi$ among the studies. This introduces *J* new nuisance parameters that saturate the model. The *J* parameters are reduced to one by treating $\delta _j$ as a random effect and assigning a normal distribution to it.

34 $$\begin{aligned} logit(\pi _j)&= \eta _j = \beta _0 + \delta _j \nonumber \\ \delta _j&\sim N(0, \tau ^2) \end{aligned}$$

Unlike the beta distribution, the normal distribution is non-conjugate to the binomial distribution. This does not pose any conceptual problem except that integrating out the random effects to obtain the log-likelihood function requires numerical approximation.

##### Retrieving the study weights

The conditional maximized log likelihood contains all current information about $\hat{\beta }_0$ and $\hat{\delta }_j$ through $\hat{\eta }_j$. Following the logic in equations (25), approximating the $j^{th}$ study contribution to the conditional maximized log-likelihood by a normal distribution yields

35 $$\begin{aligned} \nu ^{2}_j&= \frac{(1 + e^{\hat{\eta }_j})^2}{N_j e^{\hat{\eta }_j}} \nonumber \\&= \frac{1}{N_j \hat{\pi }_j (1 - \hat{\pi }_j)} \nonumber \\&\equiv \frac{N_j}{\hat{n}_j (N_j - \hat{n}_j)} \end{aligned}$$

where $\hat{\eta }_j = (\hat{\beta }_0 + \hat{\delta }_j)$, $\hat{\delta }_j$ is the “posterior” (also called the empirical Bayes) mean estimate of $\delta _j$, $\hat{\pi }_j = \frac{e^{\hat{\eta }_j}}{1 + e^{\hat{\eta }_j}}$ and $\hat{n}_j = N_j \hat{\pi }_j$. The weights that sum to 1 are then computed as

36 $$\begin{aligned} w^v_j&= \frac{N_j \hat{\pi }_j (1 - \hat{\pi }_j)}{\sum _{j=1}^{J} N_j \hat{\pi }_j (1 - \hat{\pi }_j)} \nonumber \\&\equiv \frac{ \frac{\hat{n}_j (N_j - \hat{N}_j)}{N_j} }{\sum _{j=1}^{J} \frac{\hat{n}_j (N_j - \hat{N}_j)}{N_j} } \end{aligned}$$

The relation between the $w^c_j$ and $w^v_j$ weights exists. When $\frac{1}{\hat{\pi }_{c}(1 - \hat{\pi }_{c})} > \frac{1}{\hat{\pi }_j(1 - \hat{\pi }_j)}$ the study is up-weighted and vice-versa.

##### Inference about the mean

Approximate inference for the parameter coefficients of the logistic regression model is based on large-sample theory. The number of studies need not be large for the large-sample approximation to be good. A quick convergence of the model is often a reassurance that the asymptotic properties of the regression coefficient estimates may be applicable. The conditional mean is

37 $$\begin{aligned} E(\pi _j|\delta _j = 0) = \frac{e^{\beta _0}}{1 + e^{\beta _0}} = \bar{\pi }_{cond} \end{aligned}$$

It represents the mean for a central study with $\delta _j = 0$ which may also be interpreted as the median proportion. An estimate of the population mean is

38 $$\begin{aligned} E(\pi _j)&= E(logit^{-1}(\beta _0 + \delta _j)) \nonumber \\&= \int _{-\infty }^{\infty }logit^{-1}(\beta _0 + \delta _j)f(\delta _j, \tau ^2)d\delta _j \end{aligned}$$

Unfortunately, the integration above does not follow any standard parametric form and is numerically approximated. The direct approach to obtain the point estimate is

39 $$\begin{aligned} \bar{\pi }_{pop}&= E(\hat{\pi }_j) = E(logit^{-1}(\hat{\beta }_0 + \hat{\delta }_j)) \nonumber \\&= \frac{ \sum _{j=1}^{J} \hat{\pi }_j}{J} = \frac{ \sum _{j=1}^{J} logit^{-1}(\hat{\beta }_0 + \hat{\delta }_j)}{J} \end{aligned}$$

where $\hat{\delta }_j$ is the empirical Bayes mean estimates of $\delta _j$. The simplest and most reliable way to generate the distribution of $\bar{\pi }_{pop}$ is by simulating the “posterior” distribution of $\hat{\pi }_j$. The following procedure inspired by Bayesian inference is applied

1. Let $\hat{\varvec{\Omega }}$ and $\hat{\varvec{\Sigma_\Omega }}$ denote the estimated model parameters and their variance-covariance matrix. Create S (1000 is sufficient) random simulations of the parameters
  40 $$\begin{aligned} \tilde{\varvec{\Omega }} \sim N(\hat{\varvec{\Omega }}, \hat{\varvec{\Sigma_\Omega }}) \end{aligned}$$
2. Let $\tilde{\varvec{\delta }}$ and $\tilde{\varvec{\Sigma }_\delta }$ denote the simulated random effects and their variance-covariance matrix. For each study, create S random simulations of the random effects using their simulated distributions
  41 $$\begin{aligned} \tilde{\varvec{\delta }} \sim N(0, \tilde{\varvec{\Sigma }_\delta }) \end{aligned}$$
3. For each study, compute the simulated proportion $\tilde{\pi }_j$ by adding its simulated fixed and the random effects, and the empirical Bayes mean estimate of the random effects together and then convert to [0, 1] scale
  42 $$\begin{aligned} \tilde{\varvec{\pi }}_j = logit^{-1}(\tilde{\varvec{\beta }} + \varvec{\hat{\delta }} +\tilde{\varvec{\delta }}) \end{aligned}$$
4. For each set of simulations, compute the population mean
  43 $$\begin{aligned} \tilde{\bar{\varvec{\pi }}}_{pop} = \frac{\sum _{j=1}^{J} \tilde{\varvec{\pi }}_j}{J} \end{aligned}$$
  We generate the distribution of $\widehat{RR}_j$ and $\widehat{OR}_j$ and any other functions from the simulated $\hat{\pi }_j$.
5. Obtain the $\alpha /2$ and $100 - \alpha /2$ percentiles from the simulated distribution of $\tilde{\bar{\pi }}_{pop}$ (or other functions of $\hat{\pi }_j$). In a nutshell, we obtain simulations of regression coefficients, then generate a “posterior” distribution of $\pi _j$ and its functions given the observed data $\varvec{Z}_j$ and the empirical Bayes estimates of the random effects.

##### Example II - A systematic review on the efficacy of cold coagulation as a treatment for cervical intraepithelial neoplasia.

We now fit the varying intercept-only logistic regression model to the Dolman et al. [57] dataset 
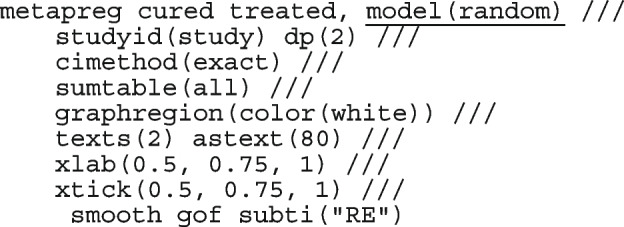


The conditional summary estimate from the model is 
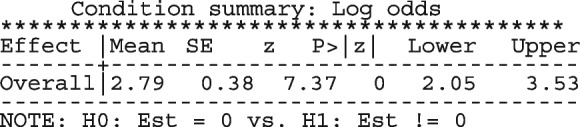
 which in the [0, 1] scale is 
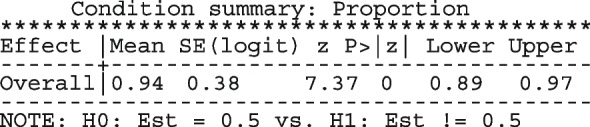


The estimate above is the mean proportion in studies with a random-effect equal to zero which also represents the median proportion. The average proportion over the whole population of the seven studies in the meta-analysis is 
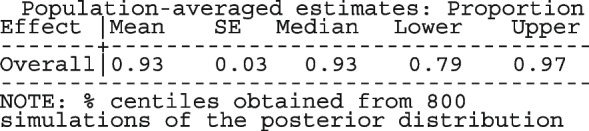


The estimate for $\tau ^2$ is 0.49 and the $I^2$ indicates that 35.04% of the total variation is unexplained. 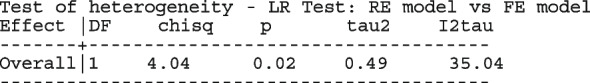


The highly significant *p*-value (0.02) from the LR test provides evidence that the model with varying intercepts is a better fit to the data. The small difference in BIC between this model (35.58) and the model with a constant intercept (36.67) indicates that the fit to the data from the two models is not very different.

The forest plot from the meta-analysis is presented in the right graph in Fig. 1. In all the studies, the model-based proportions seem consistent with the observed. By incorporating the study cure rate in the weights, the amount of information contained in the biggest study reduces from 74.03% to 58.88% moving the population-average proportion from 0.97 to 0.93.

In this dataset, the conditional summary proportion and the population-averaged proportion were not far apart. In general, the discrepancy between the two statistics increases with increase in the between-study variability $\tau ^2$.

#### Comparing independent proportions

##### Logistic regression model with varying intercepts and a constant slope

Let ($n_j, N_j, \varvec{Z}_j$) denote a data set from study *j*. The mixed effects (ME) logistic regression model for the proportion of events in study *j* is

44 $$\begin{aligned} n_j&\sim bin(\pi _j, N_j) \nonumber \\ logit(\pi _j)&= \varvec{Z}_j\varvec{\beta }^{'}_j + \delta _j \nonumber \\ \delta _j&\sim N(0,\tau ^2)\ \text {for}\ j = 1, \dots , J \end{aligned}$$

where $\beta _c$ represents the change in log odds of “success” for a unit increase in a study characteristic $Z_c$. $\beta _0$ represents the log odds of “success” when all covariates are set to 0. The omission of $\delta _j$ yields the FE logistic regression model.

##### Example III - Incomplete excision of cervical precancer as a predictor of treatment failure [59]

In 2017, Arbyn et al. [59] published a systematic review assessing the risk of therapeutic failure associated with the histological status of the margins of the tissue excised to treat cervical precancer (CIN2+). They assessed the influence of the excision procedure (cold-kife conisation (CKC), laser conisation (LC), large loop excision of the transformation zone (LLETZ), or mixed) on the margin status. They performed a stratified analysis by treatment procedure with metaprop. Their results are in column three of Table 2.

**Table 2:** Summaries from meta-analysis on efficacy of cold coagulation as a treatment for cervical intraepithelial neoplasi as perfomed by metaprop and metapreg

Treatment	Studies	metaprop	metapreg (stratified RE model)	metapreg (ME meta-regression)	metapreg (FE meta-regression)
cold-knife conisation (CKC)	17	20.17%, 95% CI[14.34, 26.71]	21.60%, 95% CI[13.44, 31.48]	21.56%, 95% CI[13.01, 25.22]	14.87%, 95% CI[14.25, 15.52]
		$\tau ^2$ = 0.10	$\tau ^2$ = 0.66		
		$I^2$ = 98.35%	$I^2$ = 94.91%		
laser conisation (LC)	13	17.76%, 95% CI[12.93, 23.17]	18.85%, 95% CI[11.46, 29.02]	18.54%, 95% CI[16.68, 37.29]	17.57%, 95% CI[16.59, 18.64]
		$\tau ^2$ = 0.05	$\tau ^2$ = 0.49		
		$I^2$ = 95.35%	$I^2$ = 89.94%		
large loop excision of the transformation zone (LLETZ)	42	25.89%, 95% CI[22.32, 29.62]	26.79%, 95% CI[21.77, - 32.28]	26.77%, 95% CI[20.79, 33.82]	29.84%, 95% CI[29.07, 30.63]
		$\tau ^2$ = 0.07	$\tau^2$ = 0.43		
		$I ^2$ = 95.76%	$I^2$ = 91.11%		
Mixed	22	23.72%, 95% CI[18.90, 28.89]	25.34%, 95% CI[17.00, 36.43]	25.26%, 95% CI[17.34, 31.33]	24.97%, 95% CI[24.07, 25.76]
		$\tau ^2$ = 0.07	$\tau^2$ = 0.99		
		$I ^2$ = 96.68%	$I^2$ = 95.55%		
All	94	23.13%, 95% CI[20.45, 25.92]	24.29%, 95% CI[20.76, 28.83]	24.34%, 95% CI[20.75, 29.53]	24.30%, 95% CI[23.84, 24.74]
		$\tau ^2$ = 0.10,	$\tau ^2$ = 0.65	$\tau ^2$ = 0.64	BIC = 3301.50
		$I^2$ = 97.63%	$I^2$ = 93.67%	BIC = 939.52	

After computing the different summary proportions, metaprop conducts a test of equality of those proportions. This test is merely an indication of the degree of evidence of no differences between the proportions but gives no information on the nature and the strength of the differences. This information can be obtained from the ratios of the proportions. The test statistics were (chi = 6.99, d.f = 3, *p*-value = 0.07) indicating no differences in the pooled proportions by treatment. In a random-effects model, the test may be biased. Two possible sources of bias are

1. The inefficiency of the MOM in estimating the between-study variance which is required in computing the weights and consequently the variances of the overall and the sub-group proportions.
2. In calculating the heterogeneity statistic, the sub-group pooled estimates are treated as though they are fixed-effects estimates while they are random-effects estimates.

To formally compare the differences between the treatment groups, we fit a ME logistic regression model with treatment as a covariate 
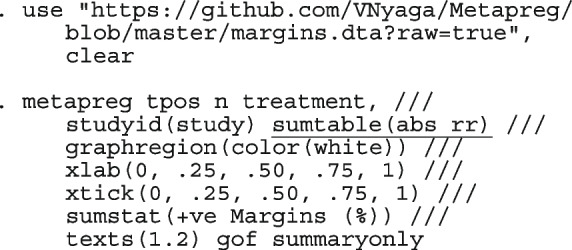


The option sumtable(abs rr) requests for the estimated positivity ratios (rr) alongside the estimated proportion (abs) of positive margins. A representation of the requested model is 
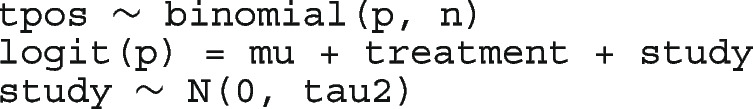


**Fig. 2:**
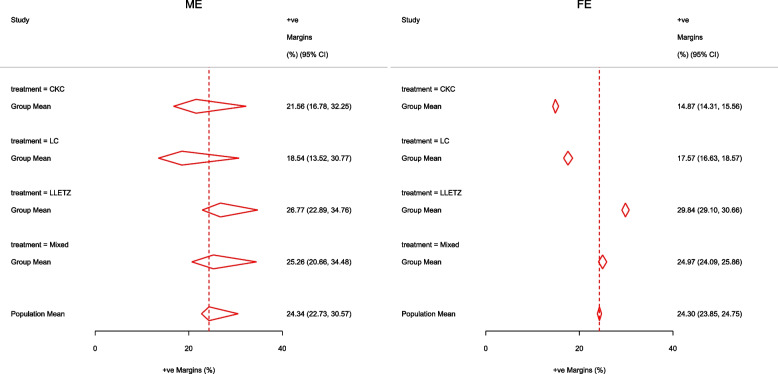
Forest plot from a meta-analysis on the incomplete excision of cervical precancer by treatment procedure (CKC - cold knife conisation, LC - laser conisation, LLETZ - large loop excision of the transformation zone) using the ME and FE logistic regression

Other outputs displayed in the results window include a representation of the mean function of the reduced model fitted for model comparison, study-specific inferences, conditional and population-averaged inferences. The estimated population-averaged proportions are presented in column five of Table 2 and in the left graph of Fig. 2. The output below indicates that large loop excision was associated with 42% higher positive margins than cold knife conisation (RR = 1.42, 95% CI[1.05, 1.93]). 
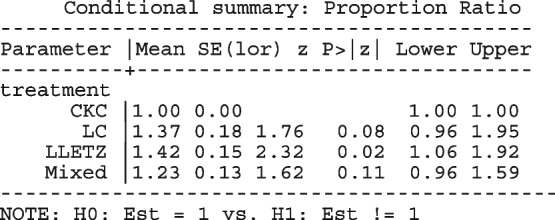

However, looking at the output below comparing all the three ratios indicates that there are no significant differences between them. 
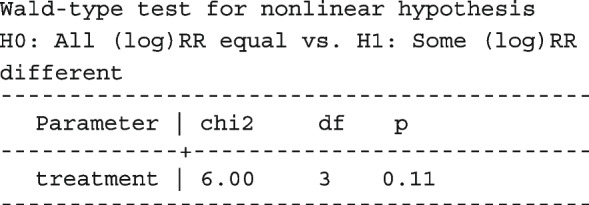


The ratios presented earlier apply to studies where the random effect is zero. By looking at the population-averaged estimates, there is no difference between large loop excision and cold knife conisation. 
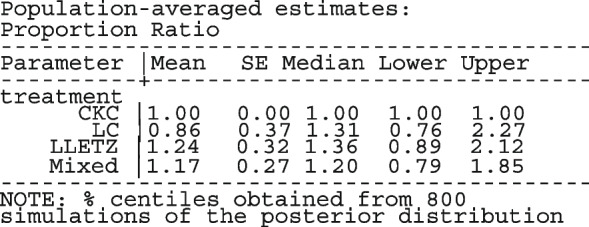


With a reduction of 7.33, the BIC indicates that the model without the covariate is more parsimonious. The *p*-value conveys the same information differently; that there no significant differences between the treatment groups. 
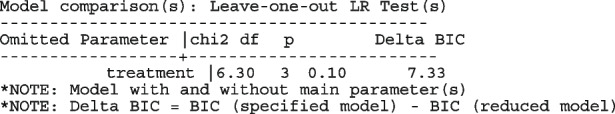


To assess the adequacy of the model, we also fitted the FE logistic meta-regression. The estimated population-averaged proportions are presented in column six of Table 2 and the right graph in Fig. 2. For comparison with the original analysis, we also performed stratified meta-analysis and fitted a RE logistic regression model for each treatment group.

In Table 2, the estimates for $\hat{\tau }^2$ from metaprop and metapreg in columns three and four should not be compared because they have different scales. However, the estimates for $I^2$ can be compared. Their differences are explained as follows. metaprop computes the $I^2$ statistic by Higgins and Thompson [60] which has been shown to lead to an incorrect conclusion of very high heterogeneity [53]. metapreg computes the $I^2$ statistic by Zhou and Dendukuri [53] which is more suitable for binomial-normal data. metaprop yields a larger estimate of the between-study variability in the CKC group $(\tau ^2=0.10)$ than in the mixed group $(\tau ^2=0.07)$ while a visual inspection of Fig. 2 suggests the opposite. In contrast, the estimates from metapreg $(\tau ^2=0.66$ vs $\tau ^2=0.99)$ are congruent with the observed variability in the forest plots (see Fig. 2); more heterogeneity in the mixed group. This discrepancy points to the statistical sub-optimality of the MOM in estimating the between-study variability [61].

The estimated population-averaged proportions from the stratified RE logistic regression and the ME logistic meta-regression models in the fourth and fifth columns are not far apart. The estimated population-averaged proportions from the ME logistic meta-regression and the FE logistic meta-regression models in the fifth and sixth columns have some discrepancies. The leave-one-out LR test from the FE model (*p*-value < 0.01) indicates differences by treatment while the RE model (*p*-value = 0.10) indicates no difference. With the lower BIC, the ME logistic meta-regression model is more parsimonious than the FE logistic meta-regression. However, the discrepancies in the results from the FE and ME suggests that the random effects may be concealing a serious model inadequacy possibly due to omission of important covariates.

#### Comparing two dependent proportions

Let $([n_{j1}, N_{j1}], [n_{j2}, N_{j2}])$ denote the paired responses from study *j*. Let $T_{1} = 0$ in the control group and $T_{2} = 1$ in the treatment group. The relative response rate is $RR_j = \frac{n_{j1}*N_{j2}}{N_{j1}*n_{j2}}$. The asymptotic score CI for $RR_j$ are computed via the Koopman [62] method which relies on an iterative likelihood optimization.

##### Logistic regression model with fixed intercepts and a constant slope - a common OR model

Under the hypothesis that all studies have the same treatment effect and produce independent estimates of the common effect, we can estimate a summary measure of the conditional association. For group *k* from study *j*, we model the proportion of positive responses as

45 $$\begin{aligned} n_{jk}&\sim bin(\pi _{jk}, N_{jk}) \nonumber \\ logit(\pi _{jk})&= \beta _0 + \theta T_k + \delta _j \nonumber \\&\text {for}\ j = 1, \dots , J \nonumber \\&\text {and}\ k = 1, 2. \end{aligned}$$

where $\theta$ is the common log odds ratio, $\beta _0$ is the log odds of a positive responses in the control group of the baseline study (the first study in the data set), and $\delta _j$ represents the log odds of a positive responses in the control group if study *j* relative to the baseline study. It follows then that $\pi _{j1}$ is the baseline risk in study *j* and $e^\theta$ is the common odds ratio. In a study *j*, the odds of positive response in the treatment group are $e^{\theta }$ times the odds in the control group. When $\theta = 0$, the proportions of positive responses are the same in the pair and there is no treatment effect. This model has as many parameters as the number of studies. Models with less parameters are preferred because yield more precise estimates [63]. Before describing models with less parameters, we fit the model to an example data set.

##### Example V - Intravenous magnesium for acute myocardial infarction [64]

Due to conflicting evidence from earlier meta-analyses and large trials, Li et al. [64] conducted a review to clarify further the effect of magnesium (versus control) on early mortality. Using the Review Manager, they fitted the CE linear model and the RE linear model to the log odds ratio. The CE linear model showed no effect (overall OR = 0.99, 95% CI[0.94, 1.04]) while the RE linear model showed a significant effect (overall OR = 0.66, 95% CI[0.53, 0.82]). In an attempt to explain the variation in the treatment effects among the studies, they performed subgroup meta-analyses by time since the onset of symptoms (< 6 hours, 6+ hours), use of thrombolysis, and dosage (< 75 mmol, 75+ mmol). They concluded that the effect of treatment with low dose (< 75 mmol) and use of thrombolysis treatment on the effect of magnesium on mortality was uncertain. Based on the inference from the CE linear model, they discouraged the use of intravenous magnesium. Doi et al. [1] used this dataset to demonstrate why the IVhet linear model should replace the CE and RE linear model.

We begin by reproducing the meta-analyses with metan
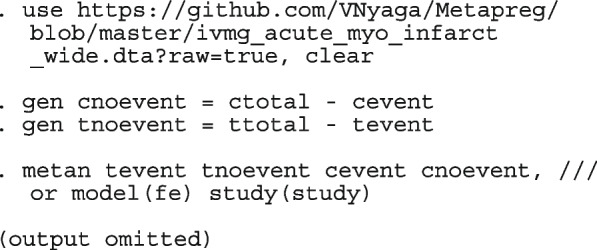


Replacing the option model(fe) with model(re) and model(ivhet) in the syntax above fits the RE and IVhet linear models, respectively.

**Fig. 3:**
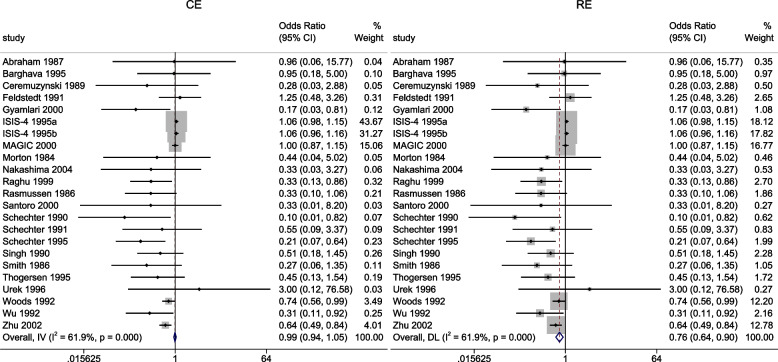
Forest plot of odds ratios of death from a meta-analysis on the intravenous magnesium for acute myocardial infarction using the stratified CE inverse-variance model and RE inverse-variance model with metan

The forest plots of the odds ratio of death from the meta-analyses using the CE and RE linear models are presented in Fig. 3. The overall OR from the CE and RE linear models were 0.99 (95% CI[0.94, 1.05]) and 0.79 (95% CI[0.68, 0.92]) respectively. As expected, the estimates from the IVhet model are identical to the CE model but with wider CIs (0.99, 95% CI[0.79, 1.24]). The overall OR in Doi et al. [1] from the IVhet and the RE models were 1.01(95% CI[0.71, 1.46]) and 0.71 (95% CI[0.57, 0.89]) respectively. Their results differ from ours in two ways. First, they combined the stratified data from the ISIS-4. Secondly, they excluded three studies from their analysis (two with zero cells). The naive SE from the CE linear model and the corrected/robust SE from the IVhet linear model were 0.025 and 0.11, respectively. Generally, a substantial difference between the naive and corrected SEs, as is in this case, calls for more attention in modeling of the mean and correlation structure.

Li et al. [64] argued that neither the CE nor the RE linear model analysis were appropriate and that a Bayesian perspective could help reconcile the discordant ISIS-4 findings from the other trials. Model parameters estimated in the Bayesian and the frequentist perspective will not differ unless informative priors are used. Using metapreg, we will show that the results are different because the model assumptions are different. The code to fit the constant-baseline logistic regression model is 
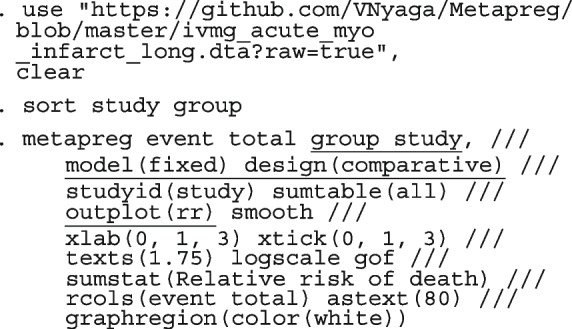


The fitted model has group and study as covariates 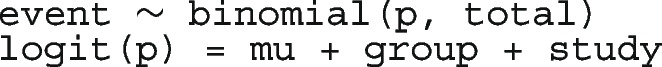


The estimated common log OR (-0.01, 95% CI [-.07, 0.04]), population-averaged OR (0.99, 95% CI [0.93, 1.04]), population-averaged RR (0.99, 95% CI [0.94, 1.04]) and the LR statistic (chi2 = 0.23, *p*-value = 0.63) for $H_0$ : group effect = 0 all indicate no treatment effect. 
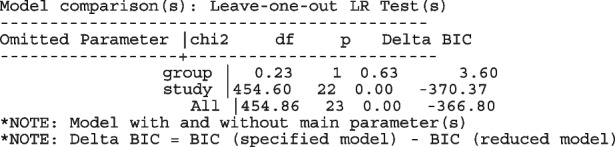


**Fig. 4:**
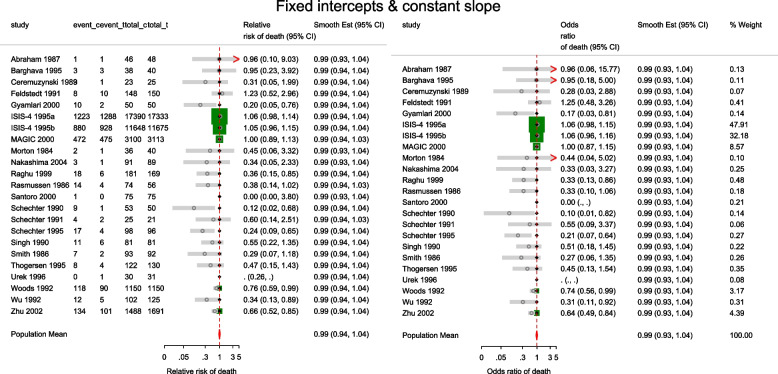
Forest plots of relative risk and odds of death from a meta-analysis on the intravenous magnesium for acute myocardial infarction using a fixed-effects logistic regression model with fixed intercepts and a constant slope

The estimated population-averaged RR is similar to the OR estimates from metan’s and the original CE models. When the event rate is rare (< 10%), as is for many studies in this data set, ORs and RRs are very similar. Generally, the OR exaggerates the effect size but when there is no treatment effect, both OR and RR are equal to 1. In the syntax, the option outplot(rr) requested for a forest plot of the probability ratios. Changing the option to outplot(or) would display of the odds ratios instead. The forest plots of the relative risk of death and the relative odds of death are presented in left and right graph of Fig. 4.

##### Logistic regression model with random intercepts and a constant slope - a common OR model

Since the main objective is to make valid and efficient inference about the average treatment effect over the population of studies and not about the incidental estimates of the baseline log odds, it makes sense to simplify model (45) by treating the baseline parameters $\varvec{\delta }$ as normally distributed random effects with mean baseline log odds $\mu _\delta$ and variance $\tau ^2_\delta$. With three parameters, the model is more manageable and can also be easily extended to incorporate study-level covariates $\varvec{Z}_j$ - possible sources of heterogeneity. The ME logistic regression model comparing the proportions of positive responses in the two groups is expressed as

46 $$\begin{aligned} n_{jk}&\sim bin(\pi _{jk}, N_{jk}) \nonumber \\ logit(\pi _{jk})&= \theta T_k + Z_j\beta ^{'} + \delta _j \nonumber \\ \delta _j&\sim N(\mu _\delta ,\tau ^2_\delta ) \nonumber \\&\text {for}\ j = 1, \dots , J \nonumber \\&\text {and}\ k = 1, 2. \end{aligned}$$

The shared random effect between the paired responses guarantees the within-study comparison between the treatments. As $\tau ^2_\delta \rightarrow 0$ the baseline risks become more homogeneous. A large $\tau ^2_\delta$ indicates high heterogeneity in baseline risks between studies and a strong association between the paired responses. 
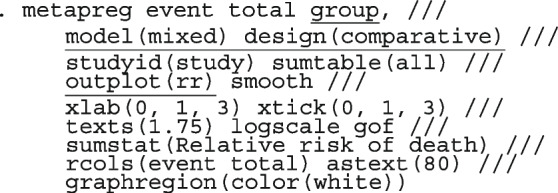


The fitted model is 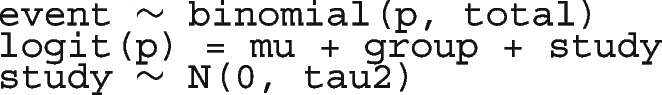


The unexplained variation of the baseline risks is estimated at $\hat{\tau }^2_\delta$ = 0.22. The reported LR test shows that the variability between the baseline risks is significant. 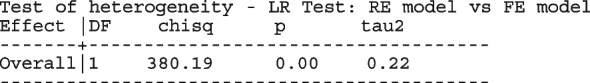


The estimated conditional and population-averaged OR, and RR are all 0.99, 95% CI [0.94, 1.04], like from the previous FE logistic regression.

**Fig. 5:**
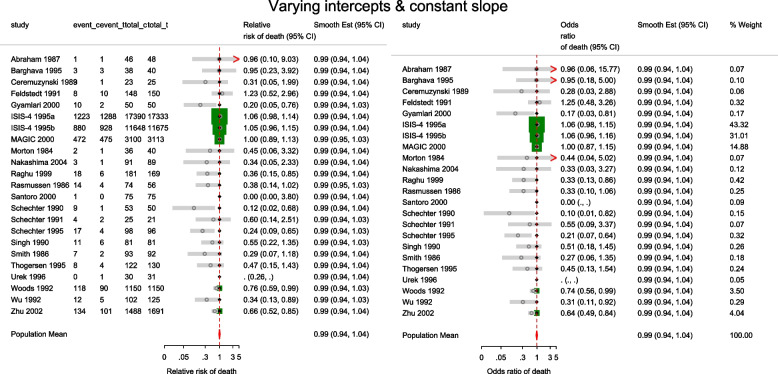
Forest plots of relative risk and odds of death from a meta-analysis on the intravenous magnesium for acute myocardial infarction using a mixed-effects logistic regression model with varying intercepts and a constant slope

The forest plot of the relative risk and odds ratio of death are presented in the left and right graph of Fig. 5. Comparing the weights displayed in Figs. 3, 4, and 5, the relatively large studies are down-weighted by the DL RE method much more than by the logistic meta-regression implicit weighting scheme. In Figs. 4 and 5, the 95% CIs of the observed RRs and ORs in Santoro 2000 and Urek 1996 represented by gray lines are missing because they are undefined in the log scale. Nonetheless, the study data is still used in the meta-regression without the continuity correction carried out by metan.

##### The saturated logistic regression model - fixed intercept and fixed slope

There are two indications in Figs. 4 and 5 that the models are a poor summary of the data. First, we observe that the model-based treatment effects are systematically larger than observed effect in most studies. Secondly, the CIs of the observed RR and the model-based RR (or OR) do not overlap in six studies. The inference from previous FE and the current ME logistic regression models are based on the assumption of identical odds ratio between the studies. If the treatment effects vary, there will be more variation in the data than explained by either model - presence of overdispersion. Furthermore, it can lead to practical problems by creating false inferences about the treatment effect. It is therefore, essential to know if the data supports the assumption of homogeneous log odds ratio. To test the hypothesis of equal odds ratio, we need to test whether the 22($J-1$) parameters in the saturated model that are coefficients of interaction terms between study and group all equal 0.

We fit the saturated model to the myocardial infarction [64] data set as follows 
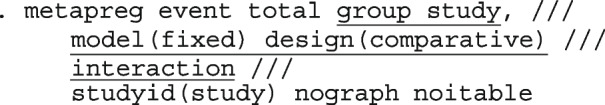


The options nograph and noitable suppress the forest plot and the table output. The fitted model now includes the interaction terms study*group
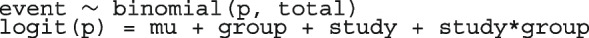


The LR test statistic for $H_0 :$ all study*group = 0 (chi2 = 68.68, *p*-value = 0.00) does not support the hypothesis of equal odds ratios. 
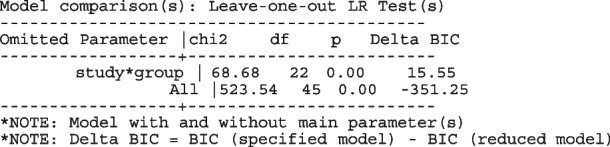


##### Logistic regression model with varying intercepts and varying slopes

To allow for varying log odds ratio by study, we introduce *J* new parameters $\theta _j$ representing the study log odds ratio - normally distributed random effects with mean log odds ratio $\mu _\theta$ and variance $\sigma ^2_\theta$. In study *j*, the proportion of events in group *k* is modelled as

47 $$\begin{aligned} n_{jk}&\sim bin(\pi _{jk}, N_{jk}) \nonumber \\ logit(\pi _{jk})&= \theta _j + Z_j\beta ^{'} + \delta _j \nonumber \\ \delta _j&\sim N(\mu _\delta ,\tau ^2_\delta ) \nonumber \\ \theta _j&\sim N(\mu _\theta ,\sigma ^2_\theta ) \nonumber \\&\text {for}\ j = 1, \dots , J \nonumber \\&\text {and}\ k = 1, 2. \end{aligned}$$

When $\sigma ^2_\theta$ is close to zero, all studies have the same treatment effect and same standard error. The total between-study variation is $(\tau ^2_\delta + \sigma ^2_\theta )$. The proportions of the variability accounted for by differences in the baseline risks and treatment effects are computed as $I^2_\delta = \frac{\tau ^2_\delta }{(\tau ^2_\delta + \sigma ^2_\theta )}$ and $I^2_\theta = \frac{\sigma ^2_\theta }{(\tau ^2_\delta + \sigma ^2_\theta )}$, respectively.

The code to fit this model to the myocardial infarction [64] data set is 
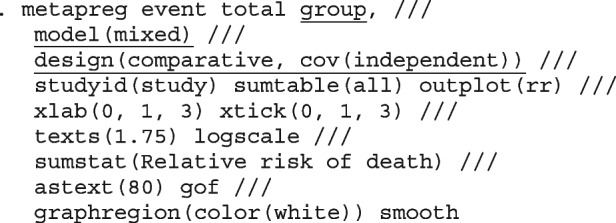


The option cov(independent) indicates that the two random effects in the model are independent. The fitted model is 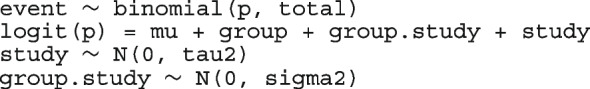


The population-averaged OR and RR are 0.69 (95% CI [0.53, 0.89]) and 0.70 (95% CI [0.56, 0.89]), respectively. Both statistics are similar to the estimate from the original RE linear model. 72.29%(I2tau) of the unexplained between-study variation is due to differences in the underlying risk of patients and 27.71%(I2sigma) is due to differences in treatment effects. 
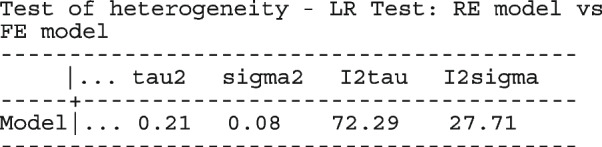


**Fig. 6:**
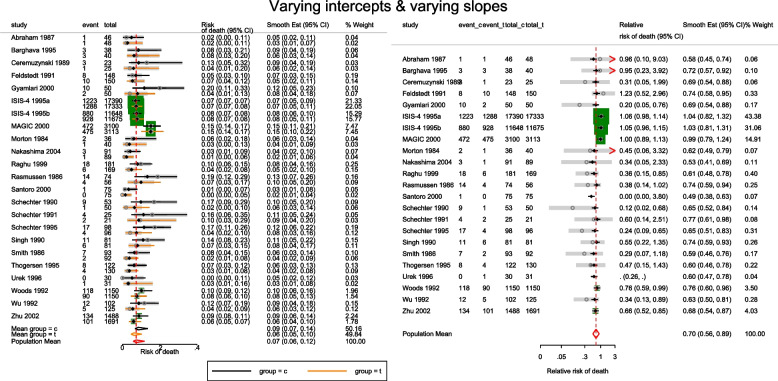
Forest plots of absolute and relative risk of death from a meta-analysis on the intravenous magnesium for acute myocardial infarction using a mixed-effects logistic regression model with varying intercepts and varying slopes

The forest plots of the absolute and relative risk of death are presented in the left and right graph of Fig. 6. Some of the 95% CIs of the observed RRs and their expected estimates do not overlap indicating that the model fits those data points poorly.

##### Logistic regression model with correlated intercept and slope

Often, the mean structure is of primary interest and not the covariance structure. Nonetheless, an inappropriate covariance structure may lead to incorrect interpretation of the variation in the data and invalidate inference for the mean structure.

To investigate whether the treatment benefit is related to the underlying risk of the patients in the different studies, we allow a correlation between the baseline risks and the treatment effect in model (47) so that

48 $$\begin{aligned} \left( \begin{array}{c} \delta _j \\ \theta _j \end{array}\right) \sim \left[ \begin{array}{cc} \tau ^2_\delta &{} \rho \\ \rho &{} \sigma ^2_\theta \end{array}\right] \end{aligned}$$

The code to fit model (47) with correlated baseline and treatment effects to the myocardial infarction [64] data set is 
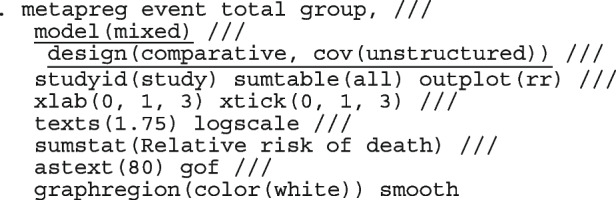


**Fig. 7:**
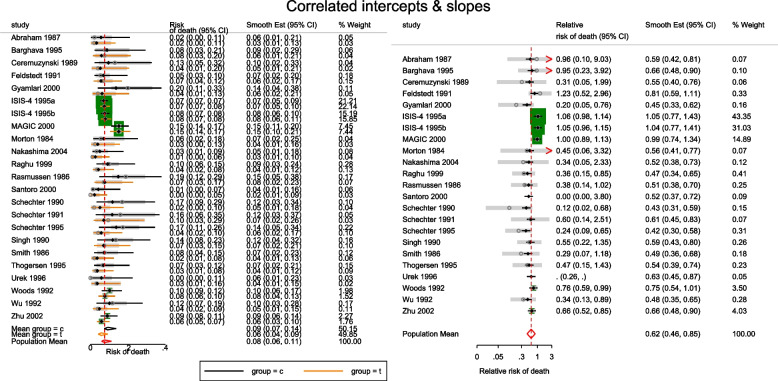
Forest plots of absolute and relative risk of death from a meta-analysis on the intravenous magnesium for acute myocardial infarction using a mixed-effects logistic regression model with correlated intercepts and slopes

The population-averaged OR and RR are 0.60 (95% CI [0.43, 0.84]) and 0.62 (95% CI [0.46, 0.85]), respectively, indicating a treatment effect. The estimated standard deviation of the baseline log odds is $\hat{\tau }_\delta = 0.52$, accounting for 72.77% of the total unexplained between-study variation. The estimated standard deviation of the log OR is $\hat{\sigma }_\theta = 0.20$ for the remaining between-study variation. The estimated correlation between the baseline risks and the treatment effect is $\hat{\rho } = - 0.77$ implying that studies with low baseline risks had a larger treatment effect size and vice-versa. The forest plots of the absolute and relative risk of death are presented in the left and right graph of Fig. 7.

##### Model checking

To place more confidence that a chosen model structure is close to the optimal model, we study the patterns in the observed RRs and compare them with the model-based estimates. The BIC reduces from 324.26 to 322.53 indicating a slight improvement to the fit. The model-based RRs represented by the solid dot and lines and the observed RRs represented by the gray dots and lines are more consistent in the unstructured covariance (see Fig. 6) than in the independence covariance (see Fig. 7). Nonetheless, the CIs of the observed and overall RRs from ISIS-4 and MAGIC studies do not overlap. A formal method for dealing with isolated discrepancies include adding dummy variable taking the value one for the discrepant study and zero elsewhere [65] and conducting an LR test to examine the effect of the discrepant studies on the fit. The addition of the dummy variable has an effect on the fit equivalent to removing the discrepant study from the data set. 
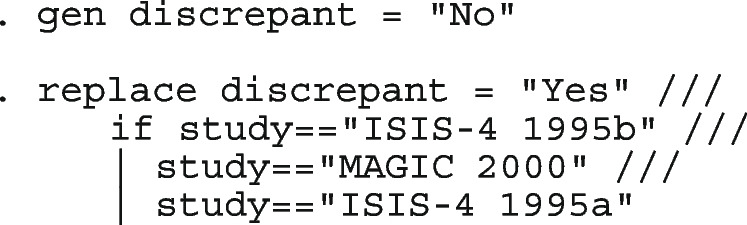


We re-fit the previous model with correlated baseline and treatment effects, and include discrepant as a potential effect modifier 
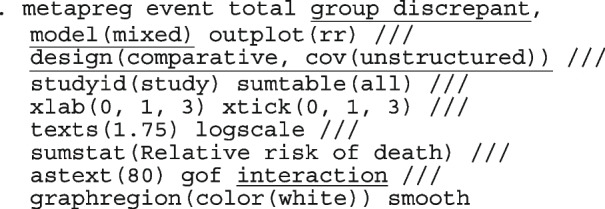


The re-fitted model is 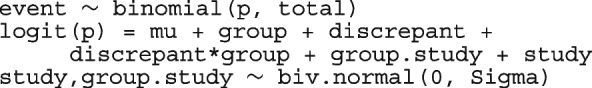


The variation in the treatment group (sigma2) disappears and the unexplained variation of the baseline risks is estimated at $\hat{\tau }^2_\delta$ = 0.23. 
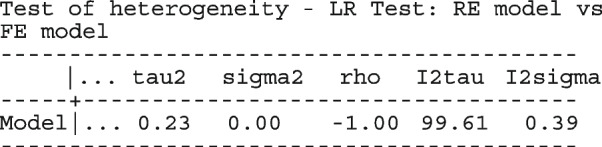


Therefore, we can drop the unnecessary parameters $\rho$ and $\sigma ^2_\theta$ from the model by removing the option cov(unstructured) in design(comparative, cov(unstructured))
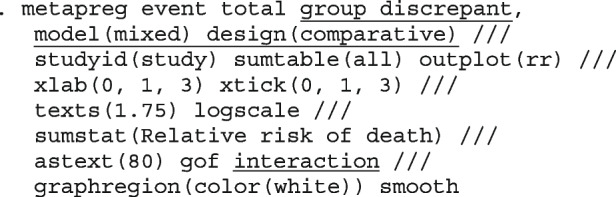


The re-fitted model is 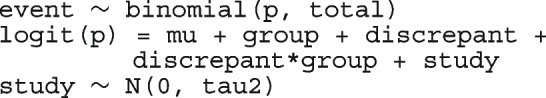


The unexplained variation of the baseline risks is estimated at $\hat{\tau }^2_\delta$ = 0.19. The estimated proportion of responses in the control group of the larger and smaller trials are not different (Yes|c = 0.10 [0.05, 0.19] vs No|c = 0.09 [0.07, 0.13]). However, the estimated proportion of responses in the treatment group was double (Yes|t = 0.10 [0.05, 0.20] vs No|t = 0.05 [0.04, 0.08])
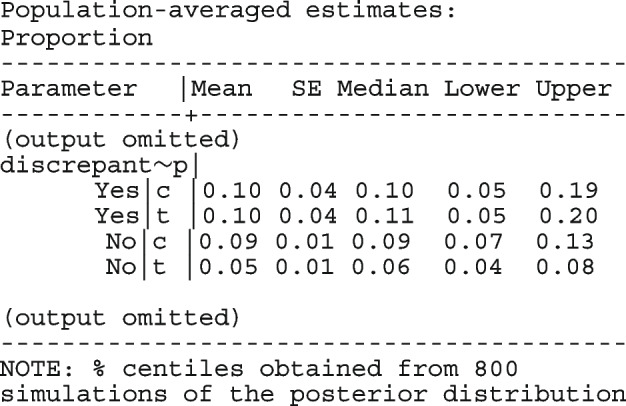


**Fig. 8:**
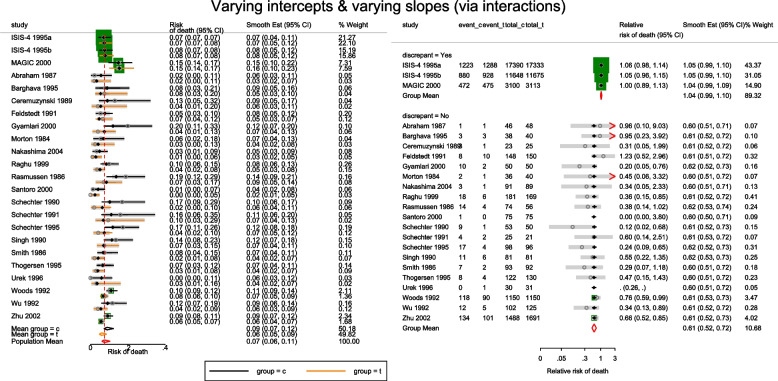
Forest plots of absolute and relative risk of death from a meta-analysis on the intravenous magnesium for acute myocardial infarction using a mixed-effects logistic regression model with varying intercepts and varying slopes

The forest plots of the absolute and relative risk of death are presented in the left and right graph of Fig. 8. The population-averaged OR and RR in the larger and smaller trials were 1.05 (95% CI [0.99, 1.11]) and 0.59 (95% CI [0.49, 0.70]), and 1.04 (95% CI [0.99, 1.10]) and 0.61 (95% CI [0.52, 0.71]), respectively. The results would barely change if we re-fitted the equivalent FE logistic regression model. metapreg cannot fit this model but it can be fitted directly into Stata. To do this, we first turn the string variables into factor variables 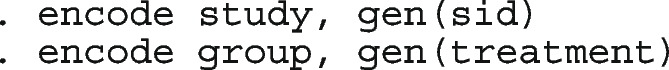

We then create two dummy variables; exception taking the value one in the discrepant studies and zero elsewhere, and rest taking the value zero in the discrepant studies and one elsewhere, and a constant x taking the value one in all studies. 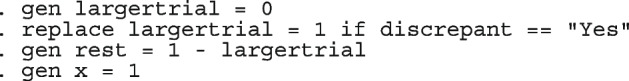


The code to fit the FE logistic regression model with constant baseline and common OR varying by discrepant group is 
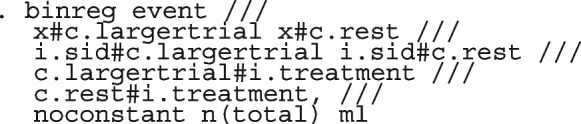


The conditional OR in the discrepant studies and the rest are 1.05 (95% CI [0.99, 1.11]) and 0.59 (95% CI [0.49, 0.68]), respectively. 
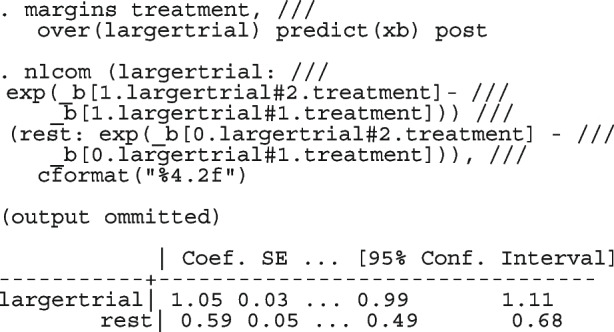


The corresponding RR estimates are 1.04 (95% CI [0.99, 1.10]) and 0.60 (95% CI [0.51, 0.70]), respectively. These are obtained as follows 
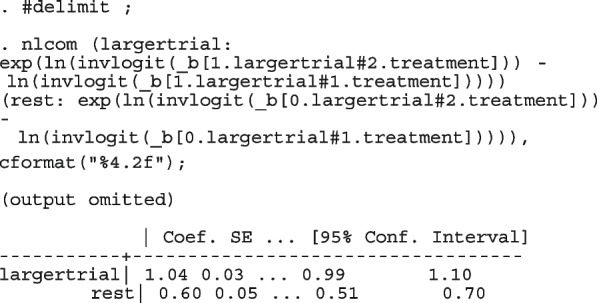


The results from the FE and ME do not differ. However, their BICs differ; their values are 304.29 and 299.99, respectively, indicating that the ME parameterisation is parsimonious. Looking at the goodness-of-fit statistic BIC in Table 3, the last ME model provides the best fit. In Fig. 8, the model-based estimates are more consistent with the observed proportions in all studies.

**Table 3:** Comparison of fixed and mixed effects models

Model	Type	BIC
Constant baseline & common slope	FE	343.12
Varying baseline & common slope	ME	337. 13
Constant baseline & varying slope	FE with interaction	358.67
Uncorrelated baseline & slope	ME	324.26
Correlated baseline & slope	ME	322.53
Correlated baseline & slope	ME with interaction	309.60
Varying baseline & common slope	ME with interaction	299.99
Varying baseline & common slope	FE with interaction	304.29

Since the weights are proportional to the study size, it makes sense to treat the model as a finite mixture model with a the fixed number of mixture components (equal to the number of studies) and the study weights as the mixing weights. Combining ideas from finite mixture modeling and K-means cluster analysis, we can interpret the working model as a segmentation of the population into two clusters such that within each cluster, the model-based log odds ratio are identical in each sub-population. There are two sub-populations; one showing substantial benefit and one without. 89.32% of the patients did not benefit from the treatment and only 10.68% of patients benefited. The results raise the question whether this clustering can be explained by known covariates. The common factors between ISIS-4 and MAGIC is treatment with high dose of magnesium (75+ mmol) and use of thrombolysis [64].

Since the data does not support the hypothesis of homogeneous treatment effects, making a single recommendation for the whole population based on the population-averaged estimate is inappropriate and misleading. It is also uninformative because the conditional effects are masked by the average effect over the whole population. Evidence from larger trials is widely believed to be more consistent with the to-be-expected true effect than the effects from smaller trials [66]. However, as the study size increases, so dose the potential presence of interactions (effect modifiers). If an interaction exist, the treatment effect in a specific sub-population will not generalize to the entire population. Therefore, the evidence from smaller trial should not be ignored.

##### Example IV - Effect of latitude on the protective effect of BCG vaccination against Tuberculosis [21]

Using the Stata command metareg [67], Sharp and Sterne [21] investigated the effect of absolute latitude (degrees north or south from the Equator) on the effectiveness of BCG vaccination. A WLS linear meta-regression model was fitted on the log odds ratios with latitude as a covariate. The analysis showed a significant negative association between the log odds ratio and the absolute latitude and the authors concluded that the benefit of BCG vaccination was greater in higher latitude. The dataset is also used in Stata to demonstrate using meta regress [12] to regress the log risk ratios against the mean-centered latitude.

We now fit an ME logistic meta-regression model with bcg; a categorical variable for the treatment group, and lat; a continuous variable with information on the absolute latitude. To allow the effects of BCG to vary by latitude, we also include the interaction between the two variables. 
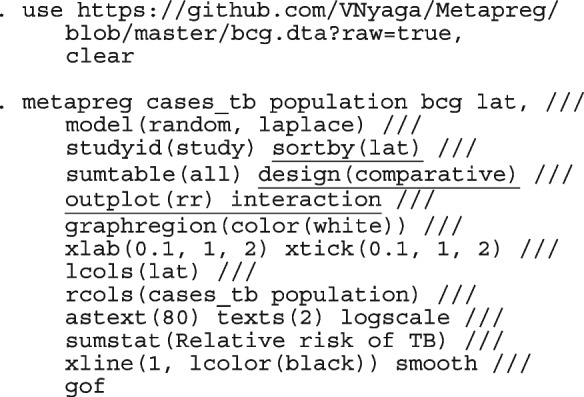


The option design(comparative) informs the procedure that the data is from a comparative study. It is therefore possible to generate a forest plot of the relative risks. The options outplot(rr) and sortby(lat) instruct the procedure to generate the forest plot (see Fig. 3) with the observed relative risks of TB sorted by lat analogous to the forest plot of the log odds ratio in Fig. 2 in Sharp and Sterne [21]. The fitted model is 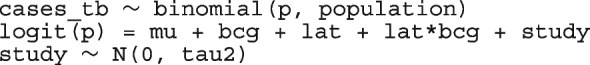


The coefficient for the interaction term lat*bcg is -0.03334 (95% CI [-0.0388, -0.02488]). This is comparable to the coefficient for lat -0.0320 (95% CI [ -0.0417, -0.0223]) when regressed against the log odds ratio of BCG vaccination reported by Sharp and Sterne [21]. The population-averaged relative risk of TB is 0.55 (95% CI [0.51, 0.60]) indicating a strong effectiveness of BCG vaccination against tuberculosis.

The significant *p*-values in the table below indicate that all the three terms (bcg + lat + lat*bcg) and especially the interaction term lat*bcg were important in explaining the variation in the risks. 
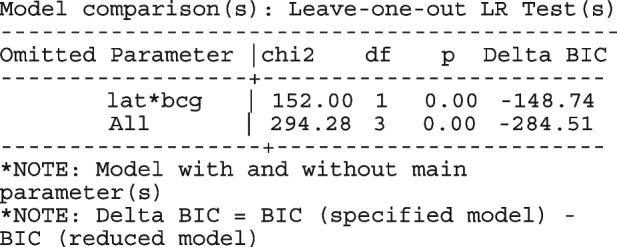


The large estimate of the between-study variance in the table below suggests omission of an important covariate. 
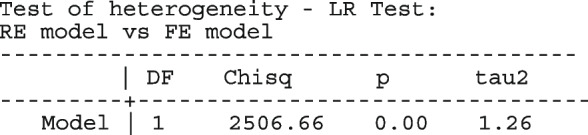


**Fig. 9:**
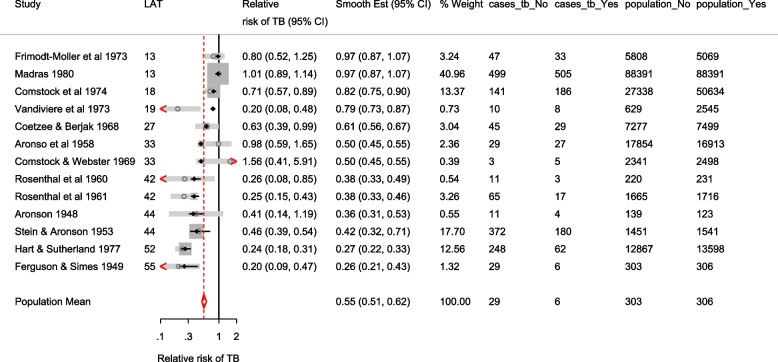
Forest plot of relative risks from a meta-analysis on the protective effect of BCG against tuberculosis using the ME logistic regression

In the forest plots presented in Fig. 9, the observed relative risks are sorted by the absolute latitude revealing a linear trend; the expected relative risk of TB decreases as the absolute latitude increases. The plot also reveals possible outlying studies. The observed and the expected risk ratios in the Vandiviere et al 1973 and the Aronso et al 1958 studies are very different indicating that the model is a poor fit for the two studies possibly due to omission of a relevant covariate.

#### Comparing multiple dependent proportions I - Contrast-based network meta-analysis

##### Logistic regression model with varying intercepts and constant slopes

Suppose each study compares at least one of *L* candidate treatments (case groups) against a comparator treatment (control group). The interest of the meta-analysis is to assess differences between the candidate treatments and estimate the average relative effectiveness of the candidate treatments relative to the comparator.

Let $(a_j, b_j, c_j, d_j)$ denote a set of case-control data from study *j* as defined in Table 4. Suppose in study *j* there are $L_j$ case groups compared to the control group, then, there will be $L_j$ tabulations as Table 4. In the dataset, this information is stored in $L_j$ rows with each row containing data from each table. From the four cells, we obtain two marginal distributions

49 $$\begin{aligned} n_{j1}&= (a_j + b_j), \nonumber \\ n_{j2}&= (a_j + c_j), \nonumber \\ N_j&= (a_j + b_j + c_j + d_j), \nonumber \\ n_{j1}&\sim bin(\pi _{j1}, N_j)\ \text {and} \nonumber \\ n_{j2}&\sim bin(\pi _{j2}, N_j). \end{aligned}$$

When a study has data on the four cells, we refer to it as a “matched” study. In many studies, only the marginal data $(n_{j1}, n_{j2}, N_j)$ is available. We refer to such data as “paired”. The terms matched and paired are often used in synonymous, but we will use them to differentiate the two data structures.

**Table 4:** Cross-tabulation of successes in the case and control group

Case	Control	
	1 (Success)	0 (Failure)
1 (Success)	$a_j$	$b_j$
0 (Failure)	$c_j$	$d_j$

Let $T_k$ be an indicator variable to distinguish the case group from the control group. We assign $T_1 = 1$ in the case group and $T_2 = 0$ in the control group. Let $F_l$ be an indicator variable to distinguish the case groups. We assign $F_l = 0$ in the first case group and $F_l = 1$ in the $l^{th}$ case group $l = (2,\dots , L)$. We propose the following model to summarize the data

50 $$\begin{aligned} n_{jkl}&\sim bin(\pi _{jkl}, N_{jkl}) \nonumber \\ logit(\pi _{jkl})&= \theta T_k + \lambda _l F_l + Z_j \beta ^{'} + \delta _j \nonumber \\ \delta _j&\sim N(0, \tau ^2) \nonumber \\&\text {for} \nonumber \\ k&= 1, 2, \nonumber \\ l&= 1, \ldots , L, \nonumber \\ j&= 1, \ldots , J. \end{aligned}$$

$\theta$ is the average log odds of “success” in the *L* case groups compared to the control group and $\lambda = (\lambda _1,\dots ,\lambda _L)$ are the case group effects. In study *j*, the odds of success in the $l^{th}$ case group are $e^{\lambda _l}$ times the odds in the $1^{st}$ case group because

51 $$\begin{aligned} \pi _{j1l}(Z_j)&= \frac{e^{(\theta + \lambda _l + Z_j\beta ^{'} + \delta _j)}}{1 + e^{(\theta + \lambda _l + Z_j\beta ^{'} + \delta _j)}}\nonumber \\&\text {and} \nonumber \\ \pi _{j11}(Z_j)&= \frac{e^{(\theta + Z_j\beta ^{'} + \delta _j)}}{1 + e^{(\theta + Z_j\beta ^{'} + \delta _j)}} \end{aligned}$$

When $\theta$ is zero, the success probability in the case and control groups are similar and when all $\lambda _l$ are zero the success probabilities between the case groups are similar.

In a forest plot, the study RR is computed as $RR_j = \frac{a_j + b_j}{a_j + c_j} = \frac{n_{j1}}{n_{j2}}$. The CIs are computed based on the score statistic [68] with constrained ML. When only the marginal summaries are available i.e. ‘paired’ data, the Koopman [62] CI’s for two independent samples are computed. These intervals are expected to be wider than the former because the intrinsic correlation in the pair is ignored.

##### Example VI - Which other hrHPV tests fulfil criteria for use in primary cervical cancer screening? [69]

To be eligible for use in cervical cancer screening, a candidate hrHPV DNA assay must fulfil three Meijer [70] criteria one of which is, it should demonstrate a relative sensitivity to detect CIN2+ compared to HC2 or GP5+/6+ PCR-EIA of $\ge$0.90. In 2015, Arbyn et al. [69] conducted a systematic review to verify which HPV tests fulfilled the three Meijer criteria.

**Fig. 10:**
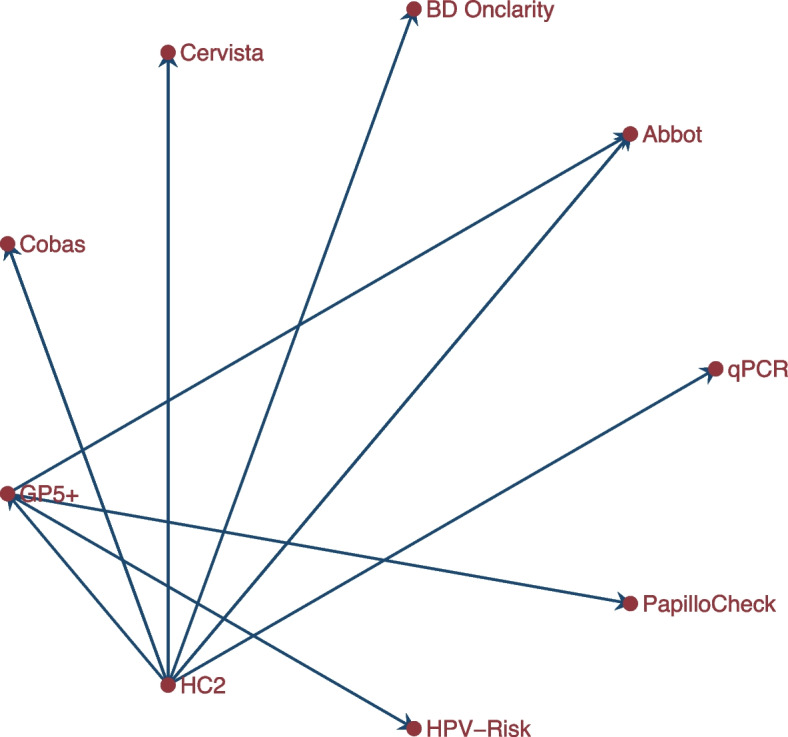
Network of eligible comparisons for the network meta-analysis of sensitivity of hrHPV DNA assays for use in primary cervical cancer screening

We focus on Table 2 in Arbyn et al. [69] The dataset included 12 studies; GP5+/6+ PCR-EIA and HC2 was the comparator in three and nine studies, respectively. Figure 10 shows a network of all the comparisons present in the data. The network plot was generated with the following code 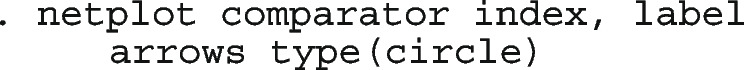

We now perform a contrast-based network meta-analysis via an ME logistic meta-regression model to verify that there are no differences in relative sensitivity among the new tests and the standard comparators. 
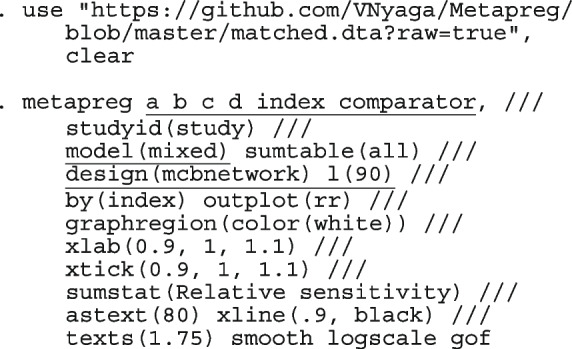


The fitted model structure is 
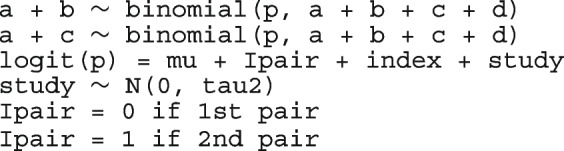


The statistics in the table below indicate that there are no differences in relative sensitivity among the new index tests and the standard comparators. 
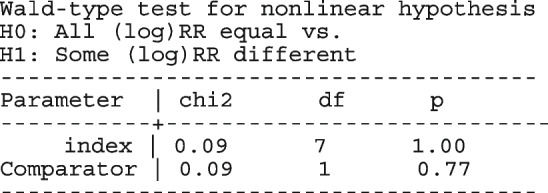


For model comparison, we also fitted the corresponding FE logistic meta-regression model (The code is not presented here). The BICs from the FE and ME models were 125.27 and 127.77 indicating that the models provide similar fits to the data. The *p*-value (0.21) from the LR test comparing the ME and FE indicates that the detected between-study heterogeneity $\hat{\tau }^2$ is not significantly different from zero. 
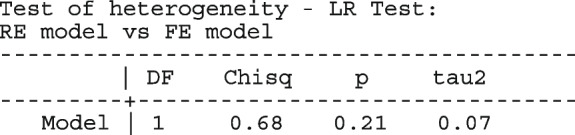


**Fig. 11:**
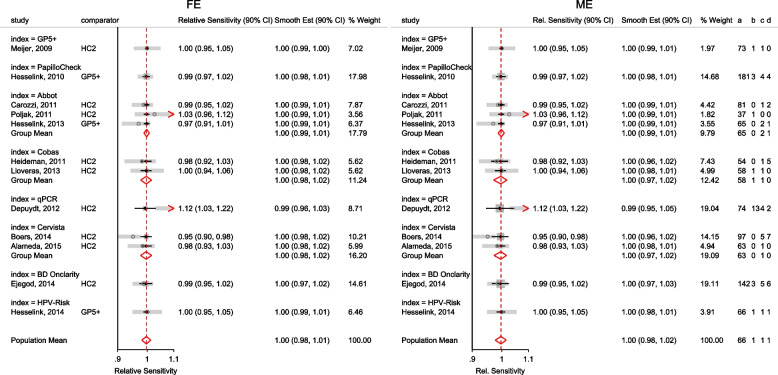
Forest plot of relative sensitivity of hrHPV DNA assays relative to HC2 or GP5+/6+ PCR-EIA from contrast-based network meta-analysis using the FE and ME logistic regression model

The forest plots from the FE and RE models presented Fig. 11 are virtually identical, as expected.

#### Comparing multiple dependent proportions II - Arm-based network meta-analysis

##### Logistic regression model with varying intercepts and varying slopes

Suppose, there are *K* treatments in total but only $K_j$ are evaluated in study *j* while the other $K-K_j$ treatments are assumed to be missing at random and interest is in summarizing the data from all the *K* different treatments coherently.

Let $([n_{j1},N_{j1}],\dots [n_{jK_j},N_{jK_j}], \varvec{Z}_j)$ denote the data from study *j*. This data is stored in $K_j$ rows with each row containing data from each treatment. We propose to model the *K* “success probabilities” as follows

52 $$\begin{aligned} n_{jk}&\sim bin(\pi _{jk}, N_{jk}) \nonumber \\ logit(\pi _{jk})&= \mu _k + Z_j\beta ^{'} + \vartheta _{jk} + \delta _j \nonumber \\ \delta _j&\sim N(0, \tau ^2_\delta ) \nonumber \\ \vartheta _{jk}&\sim N(0, \tau ^2_{\vartheta _k}) \end{aligned}$$

where $\mu _k$ is the log-odds of ‘success’ of the $k^{th}$ treatment, $(\delta _j)$ is a study effect, and $(\vartheta _{jk})$ is a treatment effect nested within a study. The imposed random-effects structure induces a positive correlation between responses from the same study $(\delta _j)$ and another between studies evaluating the same treatment $(\vartheta _{jk})$ resulting in a compound symmetry variance-covariance structure between the responses. The ICC; the correlation between any two proportions, is $\rho _\delta = \frac{\tau ^2_\delta }{\tau ^2_\delta + \tau ^2_\vartheta }$. The ICC also measures the proportion of the variability accounted for by the between-study variability. It is $\rho _\delta =0$ when the study effects convey no information and close to 1 the more identical the studies are.

To fit model 52, there should be at least 2 treatments per study for the model to be able to separate the two variance components. The specification of model 52 assumes homogeneous (equal) variance $\tau ^2_\vartheta$ between the treatments. A more flexible model allows the variances to differ by treatment i.e $\tau ^2_\vartheta = {\tau ^2_{\vartheta 1}, \dots , \tau ^2_{\vartheta K}}$ however this requires more data to identify the extra K-1 variance parameters. The model is analogous to the model by Nyaga, Arbyn and Aerts [71] for network meta-analysis of diagnostic accuracy studies.

The advantages of an arm-based network meta-analysis are potential gain in precision through the complex correlation structure, coherent inference, the possibility to combine direct and indirect evidence, and obtain any conceivable contrasts even when such contrasts do not exist from the head-to-head comparisons. Furthermore, it avoids the inflation of type I errors (multiplicity) introduced by performing a series of head-to-head comparisons [72].

##### Example VII - Comparative efficacy of antimanic drugs in acute mania [73]

**Fig. 12:**
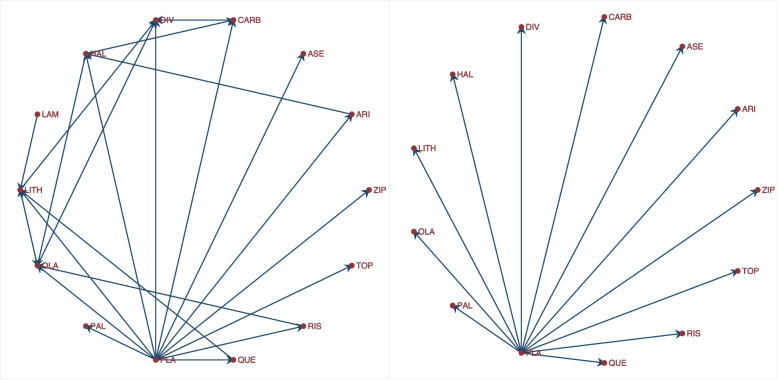
Network plot of eligible comparisons for the network meta-analysis of treatment efficacy for acute mania. The left network includes all studies while the right excludes the studies that did not evaluate the placebo

In 2011, Cipriani et al. [73] systematically reviewed 47 randomised controlled trials that compared the proportions of patients who responded to 13 treatments of acute mania in adults. The treatments included placebo (PLA), aripiprazole (ARI), asenapine (ASE), carbamazepine (CARB), valproate (VAL), haloperidol (HAL), lamotrigine (LAM), lithium (LITH), olanzapine (OLA), quetiapine (QUE), risperidone (RIS), topiramate (TOP), and ziprasidone (ZIP). Figure 12 (right) displays a network of all the comparisons in the data. From Table 5, the number of studies evaluating each treatment varied and TOP, LAM, and ASE were assessed only once. Their analysis used the Dersimonian-Laird [13] model to obtain direct evidence on the summary ORs from head-to-head comparisons of the antimanic drugs relative to placebo in Stata. They reported that all antimanic drugs were significantly more effective than the placebo except TOP. They then performed a network meta-analysis in Winbugs to obtain mixed evidence (combining direct and indirect evidence) on the summary ORs of the antimanic drugs relative to the placebo. They reported that ASE, ZIP, LAM, and TOP were not significantly more effective than the placebo. They reported further that the wide CIs from the network meta-analysis made it difficult to draw clear conclusions. In 2013, Chaimani et al. [74] used the dataset to demonstrate the use of mvmeta [11] for contrast-based network meta-analysis in Stata.

**Table 5:** Heterogeneity statistics by treatment

Treatment	Studies	*P* value (RE vs FE)	$\hat{\tau }^2$	$I^2$
ARI	7	0	0.07	73.73
PLA	36	0	0.18	78.22
HAL	8	0	0.16	60.76
QUE	7	0	0.12	76.57
LITH	8	0	0.35	70.03
ZIP	5	0	0.08	75.77
OLA	13	0	0.43	88.19
DIV	8	0	0.15	55.45
RIS	5	0	0.21	86.08
CARB	3	1	0	0
LAM	1	.	.	.
PAL	2	.	.	.
TOP	1	.	.	.
ASE	1	.	.	.

We will now demonstrate the arm-based network meta-analysis using metapreg. First, we obtain the response rates by fitting a seperate RE logistic regression model for each treatment. 
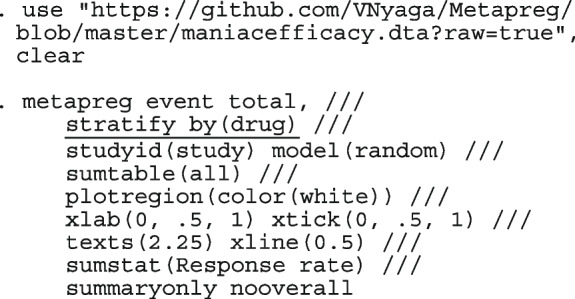


The two options stratify and by(drug) together enable us to fit separate models for each treatment group and consolidate the results in one graph and table. In contrast, the prefix by drug: would generate separate graphs and tables.

The heterogeneity statistics are presented in Table 5. The highest between-study variation was observed among studies that evaluated OLA and RIS. Some of the cells in the table for TOP, LAM, and ASE are empty because whenever there are less than three studies, the metapreg fits the CE model. Consequently, the automatic LR test comparing the RE model with the CE is not conducted. Further, the between-study variance and the $I^{2}$ are also absent. The forest plot presented in the left graph of Fig. 11 suggests that TOP was less effective than the placebo, LAM and ASE were similar to the placebo. At the same time, the other treatments were better than the placebo.

Since all studies except one included the placebo, we will perform a series of head-to-head active treatment vs. placebo comparative analyses to quantify the relative response rates. Before we fit the models, we need to put the data into the right shape for comparative analysis. This is necessary because some studies evaluated three treatments (and thus contribute three rows of data) while comparative analysis as performed with metapreg expects two rows per study. The code to reshape the data is not presented here. The right graph in Fig. 12 displays the network of treatments compared with the placebo. The network plot was generated with the following code 
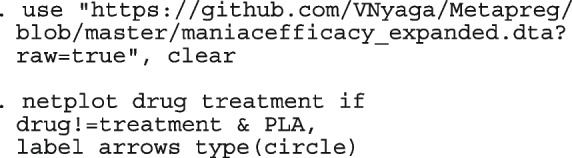


With data in the right shape, we now perform a stratified comparative meta-analyses analogous to the first analysis in Cipriani et al. [73] to obtain the direct evidence of the relative efficacy of the evaluated antimanic drugs relative to placebo. 
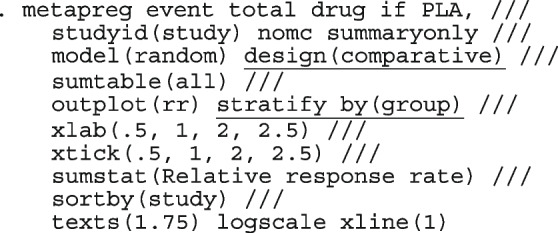


**Fig. 13:**
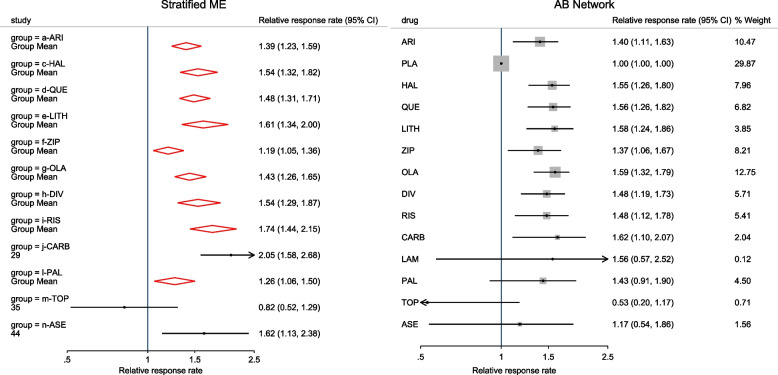
Forest plot of proportions of patients with acute mania who responded to 3-week anti-mania treatment with diverse drugs from stratified meta-analysis with RE logistic regression model (left) and arm-based network meta-analyses with ME logistic regression model (right)

For each treatment assessed in at least two studies, the fitted model structure is 
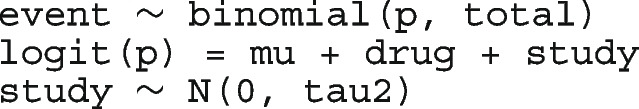
 where drug is a binary variable for treatment, either placebo or an active antimanic drug. The options design(comparative) outplot(rr) request for a forest plot of the summary response rate ratios. To save computational time we instruct the procedure not to perform model comparison with the option nomc. Otherwise, the program would also fit a logistic regression model without a drug to then perform an LR test comparing the fit of the model with and without the covariate. The summary RRs presented in the left graph of Fig. 13 indicate that all treatments were significantly more effective than placebo with the exception of TOP. These results are congruent with the conclusion by Cipriani et al. [73].

To include the information present in the data about all the comparisons between the different treatments and obtain the missing estimates for the LAM vs placebo we perform an arm-based network meta-analysis. 
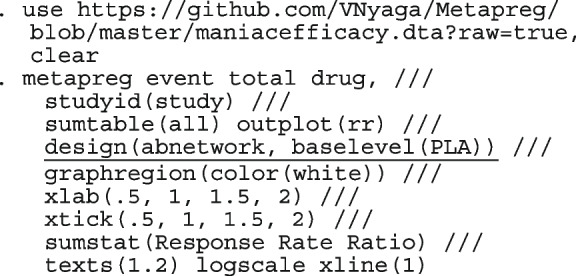
drug is a categorical variable identifying the 14 treatments. We inform the procedure that the placebo treatment is the reference category with the option design(abnetwork, baselevel(PLA).

The imposed model structure is 
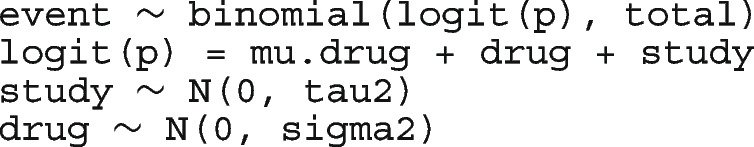


In addition to the study-specific random-effects, the model includes a second random- effect drug, a nested factor within a study.

**Fig. 14:**
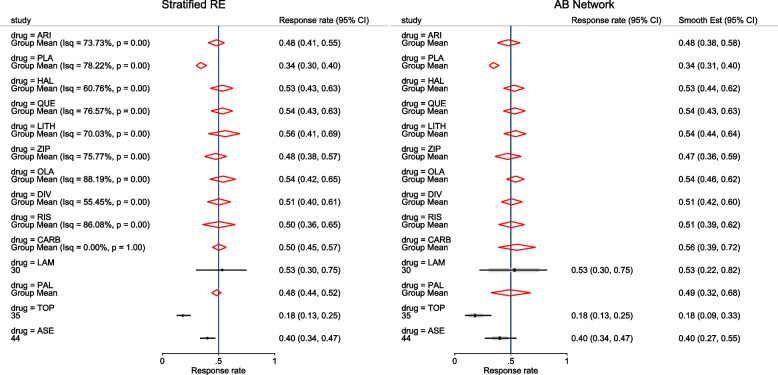
Forest plot of efficacy of antimanic drugs relative to the placebo from stratified comparative meta-analyses (left) and from arm-based network meta-analyses with ME logistic regression model (right). A relative response rate > 1 indicates higher efficacy than placebo

From the forest plot of the RR summary estimates presented in right graph of Fig. 13 , all treatments except LAM, TOP and ASE were significantly more effective than placebo. The CI’s from the arm-based network meta-analysis are narrower than the head-to-head comparisons except where a comparison was assessed in one study reflecting the uncertainty introduced by the indirect evidence. In Fig. 14, the estimated response rates from the network meta-analysis present in the right graph are consistent with those from the stratified meta-analysis presented in the left graph. The model-based response rate estimates for LAM, TOP and ASE from the network meta-analysis have shorter CIs due to borrowing information from other studies through the imposed correlation structure.

## Simulation study

To explore the utility and robustness of the logistic regression in a one-group meta-analysis of proportions, we conducted a simulation study comparing the performance of metapreg’s estimators (exact binomial (23), logistic-re-c (37), and logistic-re-m (39)) with the current RE, CE and IVhet estimators in metaprop(ftt-harm and IV) and metan (ftt-iv, ftt-geom, ftt-arith and arcsine) on the point and interval estimation of the population average proportion. No continuity correction was applied to the IV estimator in the presence of zero counts in a simulated dataset. We also included the estimates from the betabin command as the standard robust estimates. In total, we assessed 21 estimators.

We explored various scenarios in 2000 simulations, with each simulation corresponding to an independent meta-analysis. Five meta-analysis sizes were chosen *J* = 3, 5, 10, 20, and 30. The true population mean $\pi$ was set to be 0.2, 0.5 or 0.9. The sizes of each study $N_j$ were randomly chosen from a uniform distribution on the interval 10-500. Two data generation mechanisms were considered: a uniform mixture of binomials distribution and a beta mixture of binomials. To simulate the beta-binomial distribution, we first drew the binomial probabilities $p_j$ from a beta distribution with parameters *a* and *b* parameterized in terms of the population mean $\pi$ and the variance $\tau ^2$ of $\pi$

53 $$\begin{aligned} a = \frac{\pi ^2 * (1 - \pi )}{\tau ^2} - \pi \nonumber \\ b = \bigg ( \frac{\pi ^2 * (1 - \pi )}{\tau ^2} - \pi \bigg ) * \bigg (\frac{1}{\pi } - 1 \bigg ) \end{aligned}$$

Four values of $\tau ^2$ were considered for each $\pi$. Given the restriction that $a, b \ge 1$, then $0 < \tau ^2 \le \frac{1}{12}$ depending on $\pi$. The considered for values were $[\frac{1}{37.5}, \frac{1}{50}, \frac{1}{75}, \frac{1}{150}]$, $[\frac{1}{12}, \frac{1}{16}, \frac{1}{24}, \frac{1}{48}]$ and $[\frac{1}{123}, \frac{1}{164}, \frac{1}{246}, \frac{1}{492}]$, for $\pi = 0.2, 0.5$ and 0.9 respectively. Given *a* and *b*, overdispersion is $0<\phi = \frac{1}{1 + ab} \le 0.5$. $\phi$ gets larger as the spread of $\pi$ becomes wider (larger $\tau ^2$).

We assessed the estimators for bias, mean squared error (MSE, the mean of the squared bias), and empirical coverage (the percentage of meta-analyses that included the value $\pi$ in their 95% CI) in the presence of zero, weak, moderate and strong overdispersion. To compute the performance statistics of the estimators, we excluded the simulations where either the beta-binomial or logistic models failed to converge. The code to generate the simulated data can be downloaded at https://github.com/VNyaga/Metapreg/tree/master/Simulation.

## Simulation results

### Performance of the estimators in the absence of heterogeneity

The question we sought the answer to was, is the estimated heterogeneity by the RE model significantly greater than zero? The LR test between the RE and the CE model answers this question. The conundrum is that the RE model can fail to converge when the estimate for $\tau$ is on the boundary of the parameter space making it impossible to perform the LR test. With three studies, there is still 1 degree of freedom implying that the two parameters in the RE models are identifiable. The RE logistic model almost always converged while the beta-binomial model succeeded half the time (see Table 6). The RE logistic model detected significant heterogeneity in 0.90-1.01% and 1.40-1.59% of meta-analysis with three and five studies, respectively. The beta-binomial model detected significant heterogeneity twice as much; 1.84-1.98% and 2.64-3.02% in a meta-analysis of three and five studies, respectively.

**Table 6:** Convergence rate and false heterogeneity detection rate of the logistic-normal and beta-binomial model

$\pi$	Model	3 studies	5 studies
0.2	logistic-normal	99.99 (1.01)	100 (1.51)
	beta-binomial	52.04 (1.97)	53.49 (2.84)
0.5	logistic-normal	99.98 (0.90)	99.97 (1.40)
	beta-binomial	48.91 (1.84)	52.96 (2.64)
0.9	logistic-normal	100 (0.91)	100 (1.59)
	beta-binomial	47.39 (1.98)	52.47 (3.02)

**Fig. 15:**
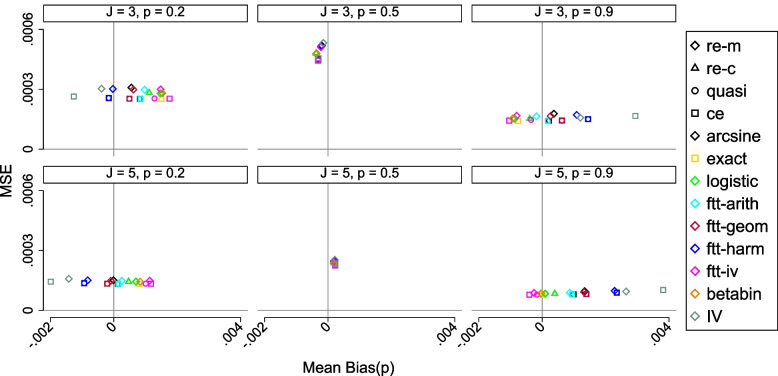
Simulation study. Scatter plot of mean squared error and mean bias of $\pi$ when $J = 3$ and 5, $\pi = 0.2, 0.5,$ and 0.9. Simulated data generated from binomial distribution. Light grey lines at 0

Figures 15 and 1 of the Supporting information show that the bias of all estimators is relatively small. When $\pi = 0.5$, the estimators yield similar estimates and differences emerge when $\pi \rightarrow 0$ or $\pi \rightarrow 1$. Over-parameterisation can lead to inefficient estimation. For this reason, the RE estimators are less efficient than the CE estimators. Increasing the size of the meta-analysis results in tremendous efficiency gain when $\pi = 0.5$.

**Fig. 16:**
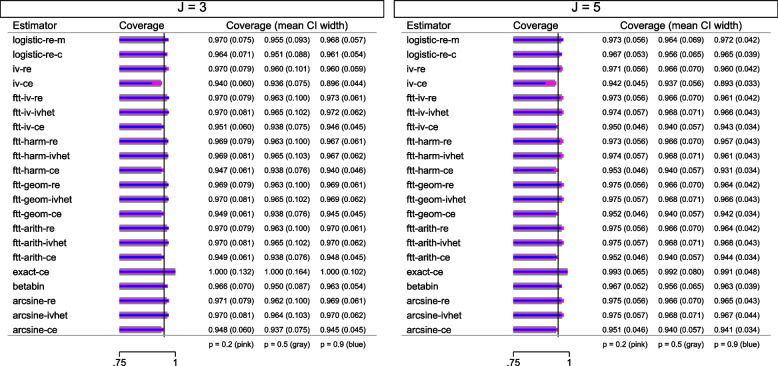
Simulation study. Empirical coverage probability of 95% confidence intervals of $\pi$ when $J = 3$ and 5, $\pi = 0.2, 0.5,$ and 0.9. Simulated data generated from binomial distribution. Light grey line at 95%

In Fig. 16, the Clopper-Pearsion CI of the exact binomial estimator is too conservative. The wald CI’s of the logistic estimators have satisfactory coverage in small meta-analyses.

### Performance of the estimators in the presence of heterogeneity

Summary statistics from a simulation study do not give the same understanding of the behavior of the estimators as graphs. Figures 2, 3 and 4 of the Supporting information show the distribution of the estimated mean for different values of the true location and variance of $\pi$ and the size of the meta-analysis. For all estimators, the distribution becomes more dispersed when $\pi = 0.5$ and when the number of studies in the meta-analysis decreases.

**Fig. 17:**
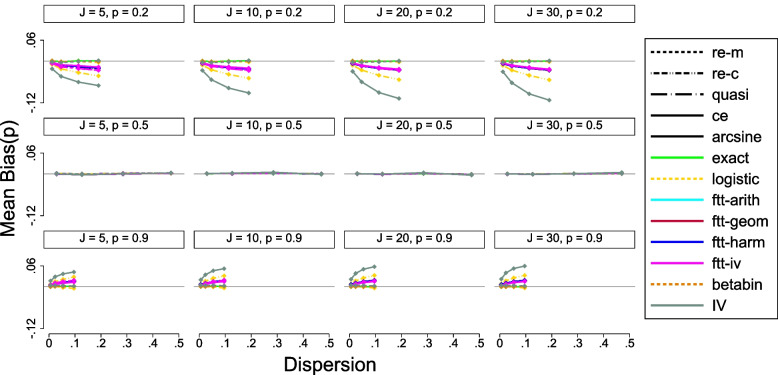
Simulation study. Bias of $\pi$. Simulated data generated from binomial distribution with 1.$\pi = 0.2$, $J = 5, 10, 20, 30$ and $\tau ^2 = [\frac{1}{37.5}, \frac{1}{50}, \frac{1}{75}, \frac{1}{150}]$ in the top panels, 2. $\pi = 0.5$, $J = 5, 10, 20, 30$ and $\tau ^2 = [\frac{1}{12}, \frac{1}{16}, \frac{1}{24}, \frac{1}{48}]$ in the middle panels, and 3. $\pi = 0.9$ and $\tau ^2 = [\frac{1}{123}, \frac{1}{164}, \frac{1}{246}, \frac{1}{492}]$ in the bottom panels. The number of studies $J = 5, 10, 20, 30$. The horizontal line at 0

Four clusters of estimators emerge in Fig. 17 with increasing bias. The exact-ce, betabin, logistic-re-m and IV-re estimators form the first-class cluster of the least biased estimators. The ftt and arcsine family of estimators form the second-class class. The logistic-re-c and the IV-ce estimators are in the third and fourth classes, respectively, with the most bias. Except in the first-class estimators, bias increases with dispersion. When $\pi \rightarrow 0$ and $\pi \rightarrow 1$, $\pi$ is underestimated and overestimated respectively.

**Fig. 18:**
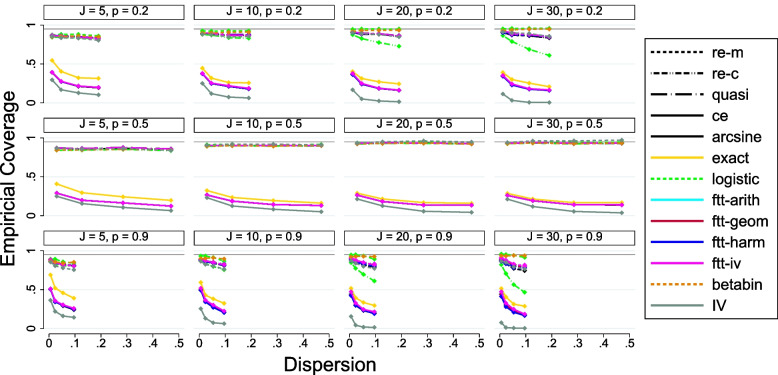
Simulation study. Empirical coverage probability of 95% confidence intervals of $\pi$. Simulated data generated from binomial distribution with 1.$\pi = 0.2$, $J = 5, 10, 20, 30$ and $\tau ^2 = [\frac{1}{37.5}, \frac{1}{50}, \frac{1}{75}, \frac{1}{150}]$ in the top panels, 2. $\pi = 0.5$, $J = 5, 10, 20, 30$ and $\tau ^2 = [\frac{1}{12}, \frac{1}{16}, \frac{1}{24}, \frac{1}{48}]$ in the middle panels, and 3. $\pi = 0.9$ and $\tau ^2 = [\frac{1}{123}, \frac{1}{164}, \frac{1}{246}, \frac{1}{492}]$ in the bottom panels. The number of studies $J = 5, 10, 20, 30$. Light grey line at 95%

Figure 18 shows that all the estimators have inferior coverage probability when the meta-analysis includes only 5 studies. Increasing the size of the meta-analysis improves the coverage of the RE and the quasi estimators except the logistic-re-c. In contrast, the coverage of the CE estimators deteriorates. Except for the beta-binomial and the logistic-re-m, the coverage of all other estimators worsens with more heterogeneity.

In Figs. 5, 6 and 7 of the Supporting information there are larger differences in efficiency when $\pi = 0.5$ compared to when $\pi$ nears its borders. All estimators lose efficiency with increasing heterogeneity. Increasing the size of the meta-analysis resulted in efficiency gain.

The bias and poor coverage of the logistic-re-c is expected. As indicated earlier, the logistic-re-c estimator the mean proportion on the condition that the random effect is zero. The discrepancy between the conditional mean proportion and the population-averaged proportion increases as $\tau ^2$ increases [75]. The poor coverage of the binomial estimator is also expected because when the structure of the mean function is correct and the true distribution is incorrect, the ML estimate of the binomial parameter will be consistent but the standard error will be incorrect [6]. The beta-bin and the logistic-re-m estimators consistently have the best coverage, least bias and highest efficiency. These results are consistent with the findings of Trikalinos et al. [76] and Lin and Chu [77].

## Discussion

The most important assumption in any statistical analysis is that the outcome measure of a choice model should accurately reflect the phenomenon of interest. However, the chosen model is never exactly correct and is only an approximation of a complex and complicated reality. The current methods of meta-analysis of binomial proportions within the approximate likelihood framework have structural flaws and suffer from loss and distortion of information. Using the binomial, logistic, or logistic-normal model for meta-analysis of binomial proportions preserves all information about the distribution $n_j \sim (\pi _j, N_j)$ from each corresponding study and models the true parameter $\pi _j$, rather than observed proportion. Consequently, obtaining functions of the model parameters is easy. This in turn enables us to obtain more detailed answers from the data(e.g. in example V). In one group meta-analysis, the binomial model assumes that nothing is known about the distribution of $\pi _j$. But this information is present in the data. In the RE logistic model the observed proportions are used as the initial information about the distribution of $\pi _j$.

The RE logistic model has been recommended [15, 41, 77] for meta-analysis of proportions. A concern often expressed about the assumption of normally distributed random effects is lack of justification [1]. In logistic regression, it is computationally convenient and natural (the variance of the binomial parameter is a function of the mean) for the random effect to enter the model on the same scale as the predictor terms. That said, the validity of any assumption regarding the random effects distribution is conceptually difficult to check because they are never directly observed. Outside meta-analysis, studies have shown through simulations that misspecifying the random-effects distribution in linear mixed-effects models [78] has negligible impact on the ML estimators. For logistic random intercept models, different assumptions for the random effects distribution often provide similar results for estimating the regression effects [79]. In meta-analyses of test performance studies, the logistic-normal model performs better than the other methods [44]. Our simulation study showed that the logistic-normal and standard robust beta-binomial model are indistinguishable in modeling overdispersed data generated from beta-binomial distribution.

For users of Stata, these models are seldom used because they have not been implemented in a user-friendly package. metapreg was expressly written and optimized for evidence synthesis of proportions from binomial proportions and could potentially improve the quality of inference. The true distribution of a random variable can never be validated. However, if a statistical model is appropriate for the observed data at hand, the behavior of the observed data should reflect the properties of the assumed distribution. A unique feature of metapreg over the existing procedures for meta-analysis of proportions in Stata is the ability to present the model-based estimates and their Wald CIs alongside the observed data. This is useful in understanding the properties of the assumed distribution, checking the model fit to the data and revealing possible outlying studies.

When should the CE or the RE logistic model be used? It is realistic and wise to be cautious and assume overdispersion is present unless and until it is shown to be absent. In true absence of heterogeneity, the RE model is over-parameterized. However, given the diversity of studies in any meta-analysis, the requirement that the expected proportion of successes is constant among the studies will be violated in a strict sense. When moving to the logistic regression with random effects, the key idea is the estimation of variation between the studies instead of testing the null hypothesis that the variance component is zero. As we have shown in the examples, the results from the RE and CE models will not be far apart unless there are important residual differences among the study results and the CE model fits the data poorly [2]. When the results from the RE and CE models are far apart, it is the task of the meta-analyst to identify important sources of variation and not doing so would be careless modeling. In general, the ME logistic regression allow us to study effects that vary by group, for example an intervention that is more effective in some studies than others (perhaps because of unmeasured study-level factors). In FE logistic regression, estimates of varying effects can be noisy, especially when there are few studies per group; ME logistic regression allows us to estimate these interactions to the extent supported by the data through.

metapreg remains a work in progress. Future research includes how to quantify the $I^2$ when there are covariates in the model and a simulation study to compare the robustness and accuracy of metapreg and the existing packages for meta-analysis of multiple binomial proportions.

The methods implemented in metapreg are statistically sound and far more robust than the current methods for meta-analysis of binomial data. To appropriately apply these methods, we recommend that meta-analysts should involve or consult a statistician with advanced statistical knowledge in meta-analysis. We expect this tutorial to accelerate the progress towards optimal methods for meta-analysis of proportions.

### Supplementary Information

**Additional file 1.**

## Data Availability

The code to reproduce the analysis herein can be downloaded at https://github.com/VNyaga/Metapreg/blob/master/metapreg-article-code.do. The code to generate the simulated data can be downloaded at https://github.com/VNyaga/Metapreg/tree/master/Simulation. Additional supporting information may be found in the online version of this article at the publisher’s web site.

## Abbreviations

QE: Quality-effects
DL: Dersimonian and Liard
GLM: Generalized linear model
GLMM: Generalized linear mixed model
ME: Mixed-effects
CE: Common-effect
FE: Fixed-effects
RE: Random-effects
ICC: Intra-class correlation
OLS: Ordinary least squares
WLS: Weighted least squares
ML: Maximum likelihood
REML: Restricted maximum likelihood
MOM: Method of moments
IV: Inverse-variance
LR: Likelihood ratio
BIC: Bayesian information criterion
IVHET: Inverse-variance heterogeneity
AIC: Akaike information criterion
CKC: Cold-kife conisation
LC: Laser conisation
LLETZ: Large loop excision of the transformation zone
CIN1: Cervical intraepithelial neoplasia of grade 1
CIN2+: Cervical intraepithelial neoplasia of grade 2 or worse
MOM: Method of moments
RR: Rate ratio
OR: Odds ratio
CI: Confidence interval
SE: Standard error
BCG: Bacille Calmette-Guérin
PLA: Placebo
ARI: Aripiprazole
ASE: Asenapine
CARB: Carbamazepine
VAL: Valproate
HAL: Haloperidol
LAM: Lamotrigine
LITH: Lithium
OLA: Olanzapine
QUE: Quetiapine
RIS: Risperidone
TOP: Topiramate
ZIP: Ziprasidone
