# Supplementary material for: Methods for meta-analysis and meta-regression of binomial data: concepts and tutorial with Stata command metapreg
Source: Arch Public Health. 2024 Jan 29;82:14. doi: 10.1186/s13690-023-01215-y (PMC10823729; doi:10.1186/s13690-023-01215-y)
Supplement: Supplementary file 1 — Additional file 1. [file 13690_2023_1215_MOESM1_ESM.pdf]

Supporting Material for Methods for meta-analysis and meta-regression of binomial proportions: concepts and tutorial with Stata command metapreg

Victoria N Nyaga and Marc Arbyn

0.1 Deriving the Maximum likelihood estimator of the exact binomial model

When it is plausible that each $\pi_j = \pi$. It follows then that

$$n_j \sim bin(\pi, N_j)$$

The density of the binomial probability distribution at n_j

$$f(n_j|\pi, N_j) = \binom{N_j}{n_j}\pi^{n_j}(1-\pi)^{N_j-n_j}$$

Therefore, the likelihood function of the CE model is

$$L(\boldsymbol{n}|\pi, \boldsymbol{N}) = \prod_{j=1}^{J}\binom{N_j}{n_j}\pi^{n_j}(1-\pi)^{N_j-n_j}$$

Ignoring the factor free of π, the jth study contribution to the log likelihood is

$$L_j(\pi) = n_j log(\pi) + (N_j - n_j)log(1-\pi)$$

Differentiating $L_j(\pi)$ with respect to π yields

$$\begin{aligned} L'_j(\pi) &= \frac{n_j}{\pi} - \frac{N_j - n_j}{1-\pi} \\ &= \frac{n_j}{\pi(1-\pi)} - \frac{N_j\pi}{\pi(1-\pi)} \end{aligned}$$

Summing $L'_j(\pi)$ over j, replacing π with $\hat{\pi}$, and equating the sum to zero yields

$$\hat{\pi} = \frac{\sum_{j=1}^{J} n_j}{\sum_{j=1}^{J} N_j}$$

Differentiating $L'_j(\pi)$ over π yields:

$$-\frac{n_j}{\pi^2} - \frac{N_j - n_j}{(1-\pi)^2}$$

Summing over j and taking the expectation, we obtain:

$$\sum_{j=1}^{J}\left(\frac{N_j\pi}{\pi^2} + \frac{N_j - N_j\pi}{(1-\pi)^2}\right) = \frac{\sum_{j=1}^{J} N_j}{\pi(1-\pi)}$$

Replacing π with $\hat{\pi}$ and taking the inverse, we obtain the asymptotic variance of $\hat{\pi}$:

$$var(\hat{\pi}) = \frac{\hat{\pi}(1-\hat{\pi})}{\sum_{j=1}^{J} N_j}$$

0.2 Simulation results

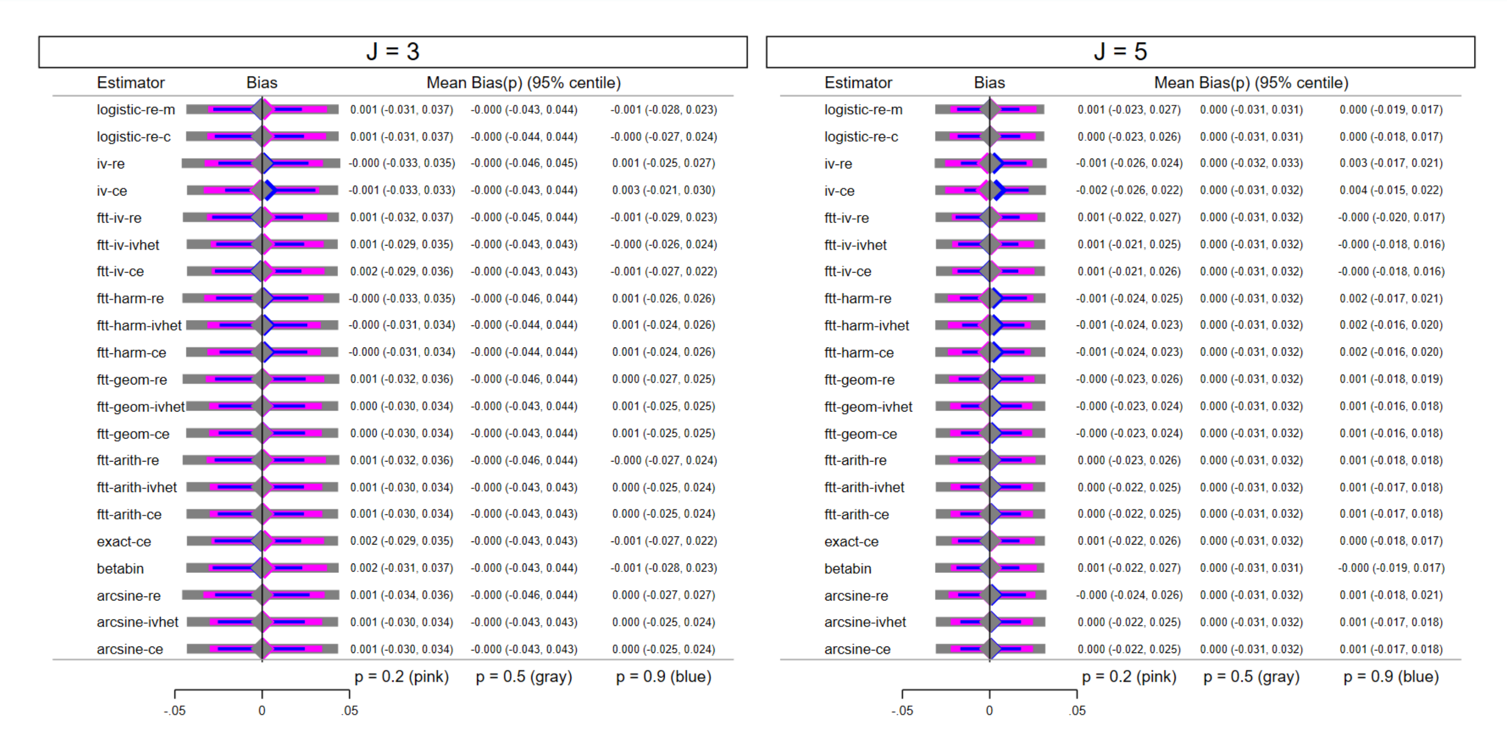

Figure 1: Simulation study. Bias of π when $J = 3, 5$ and $\pi = 0.2, 0.5, 0.9$. Simulated data generated from binomial distribution. Black line line at 0.

.

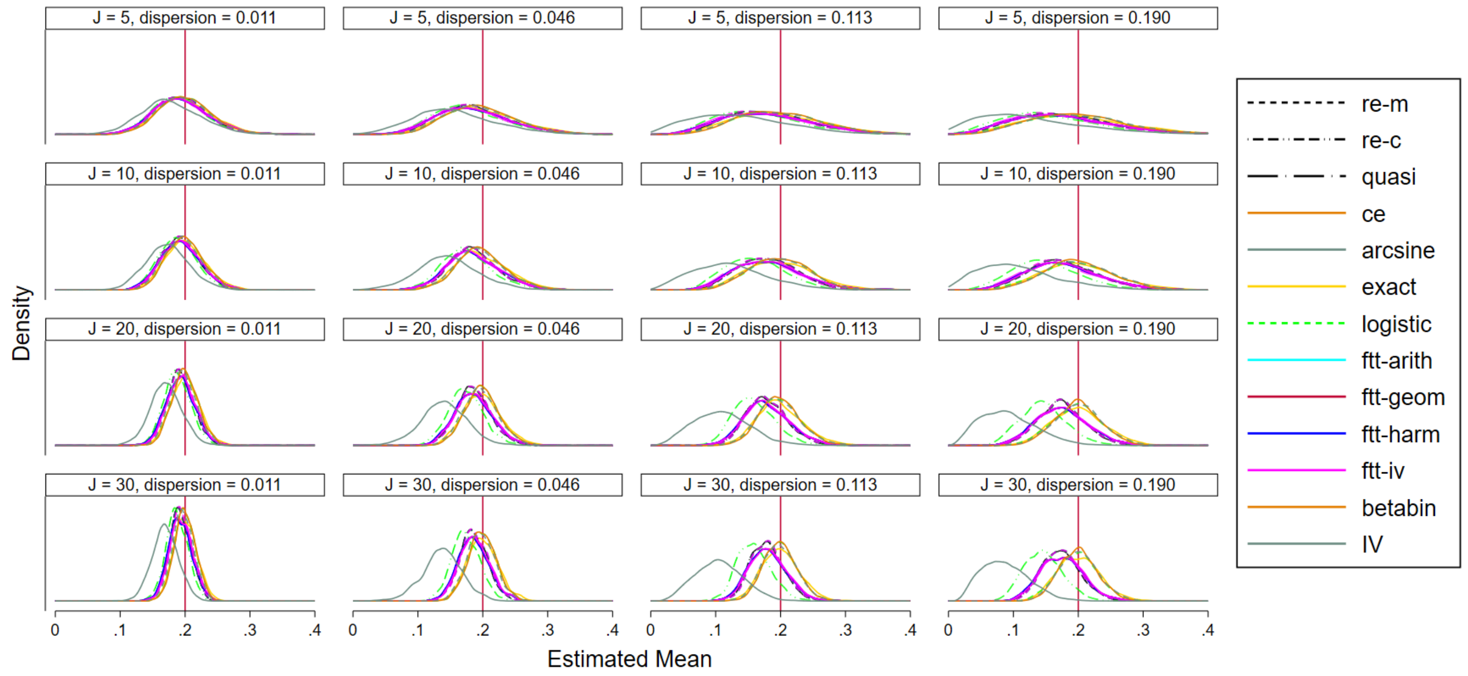

Figure 2: Simulation study. Density plots of the estimated population-averaged proportion π. Simulated data generated from binomial distribution with $\pi = 0.2$, $J = 5, 10, 20, 30$ and $\tau^2 = [\frac{1}{37.5}, \frac{1}{50}, \frac{1}{75}, \frac{1}{150}]$. The vertical bars indicate the true population value $\pi = 0.2$.

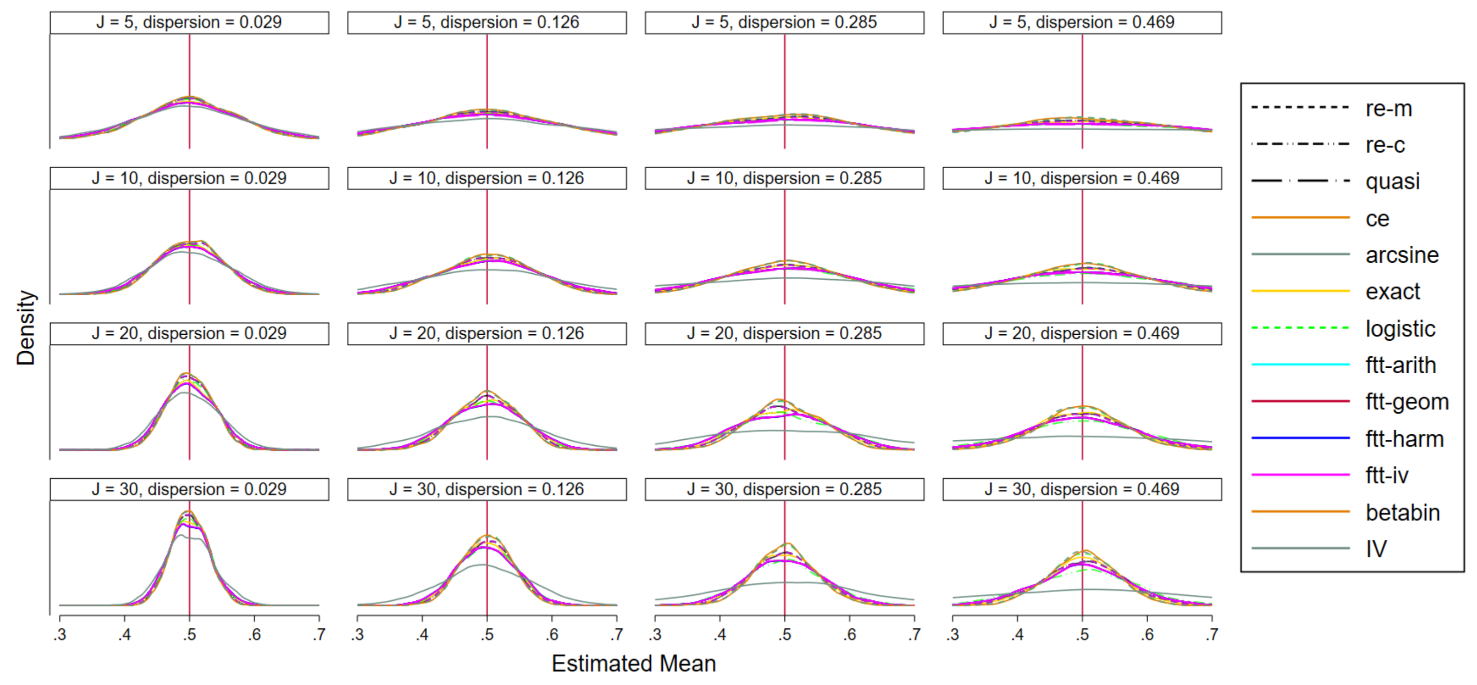

Figure 3: Simulation study. Density plots of the estimated population-averaged proportion π. Simulated data generated from binomial distribution with $\pi = 0.5$, $J = 5, 10, 20, 30$ and $\tau^2 = [\frac{1}{12}, \frac{1}{16}, \frac{1}{24}, \frac{1}{48}]$. The vertical bars indicate the true population value $\pi = 0.5$.

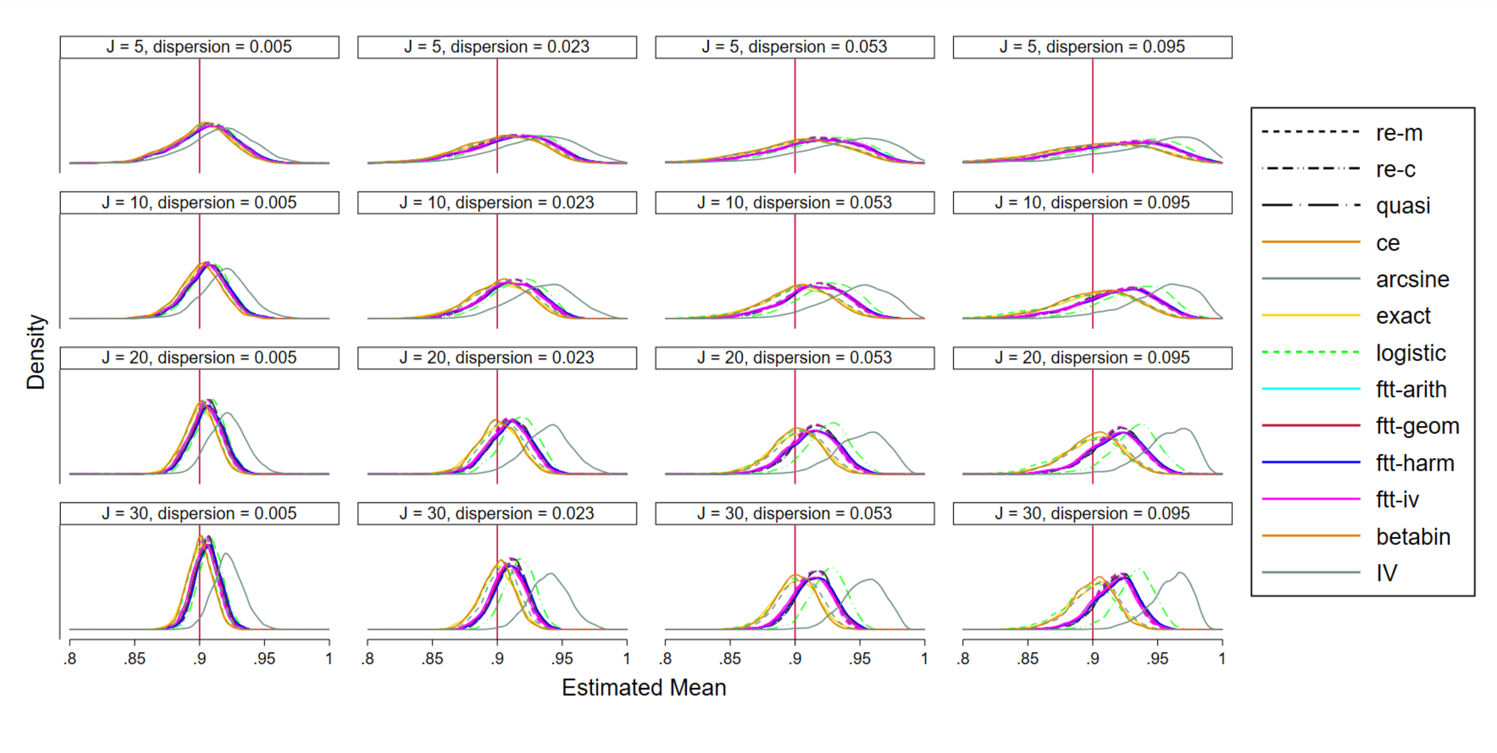

Figure 4: Simulation study. Density plots of the estimated population-averaged proportion π. Simulated data generated from binomial distribution with $\pi = 0.9$, $J = 5, 10, 20, 30$ and $\tau^2 = [\frac{1}{123}, \frac{1}{164}, \frac{1}{246}, \frac{1}{492}]$. The vertical bars indicate the true population value $\pi = 0.9$.

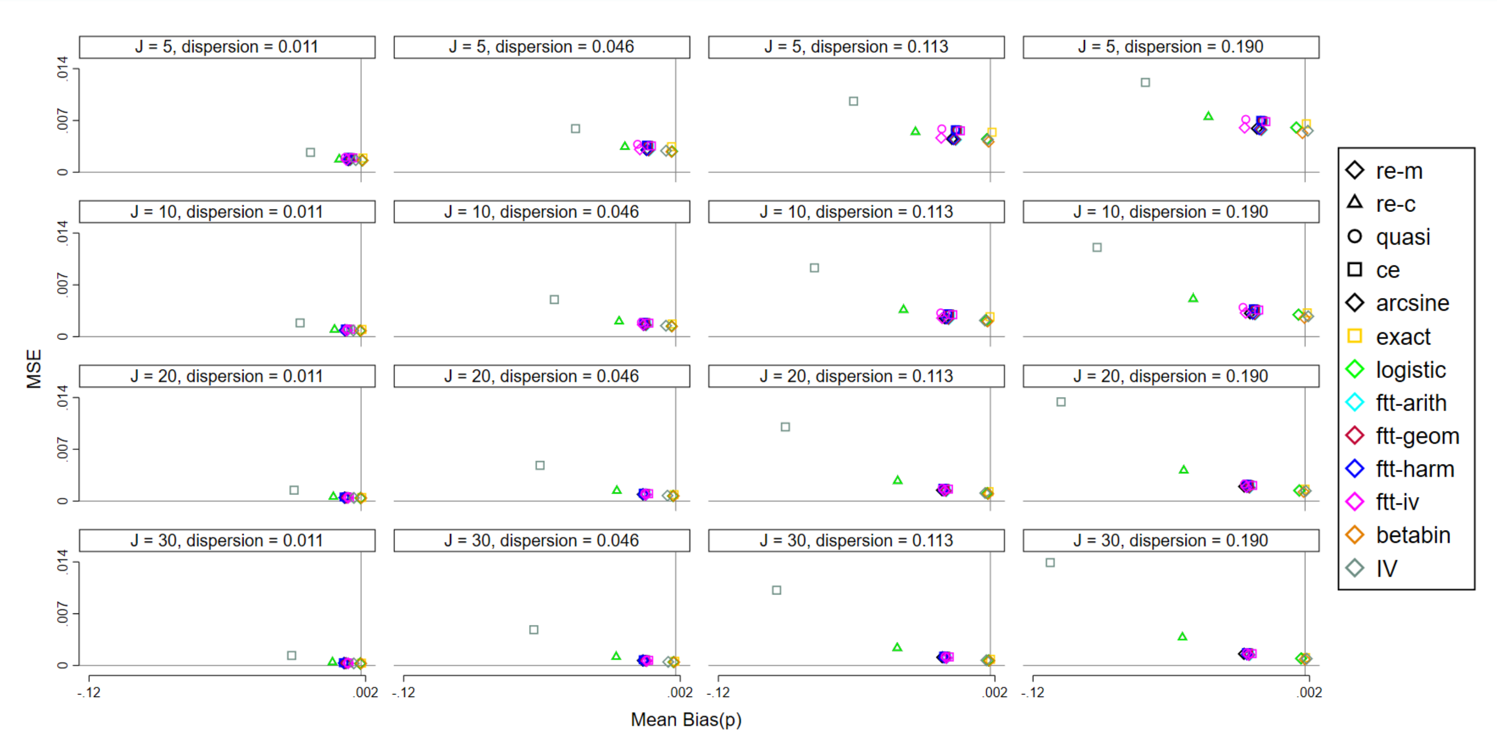

Figure 5: Simulation study. Scatter plot of mean squared error and mean bias of π. Simulated data generated from binomial distribution with $\pi = 0.2$, $J = 5, 10, 20, 30$ and $\tau^2 = [\frac{1}{37.5}, \frac{1}{50}, \frac{1}{75}, \frac{1}{150}]$. The vertical lines indicate the true population value $\pi = 0.2$.

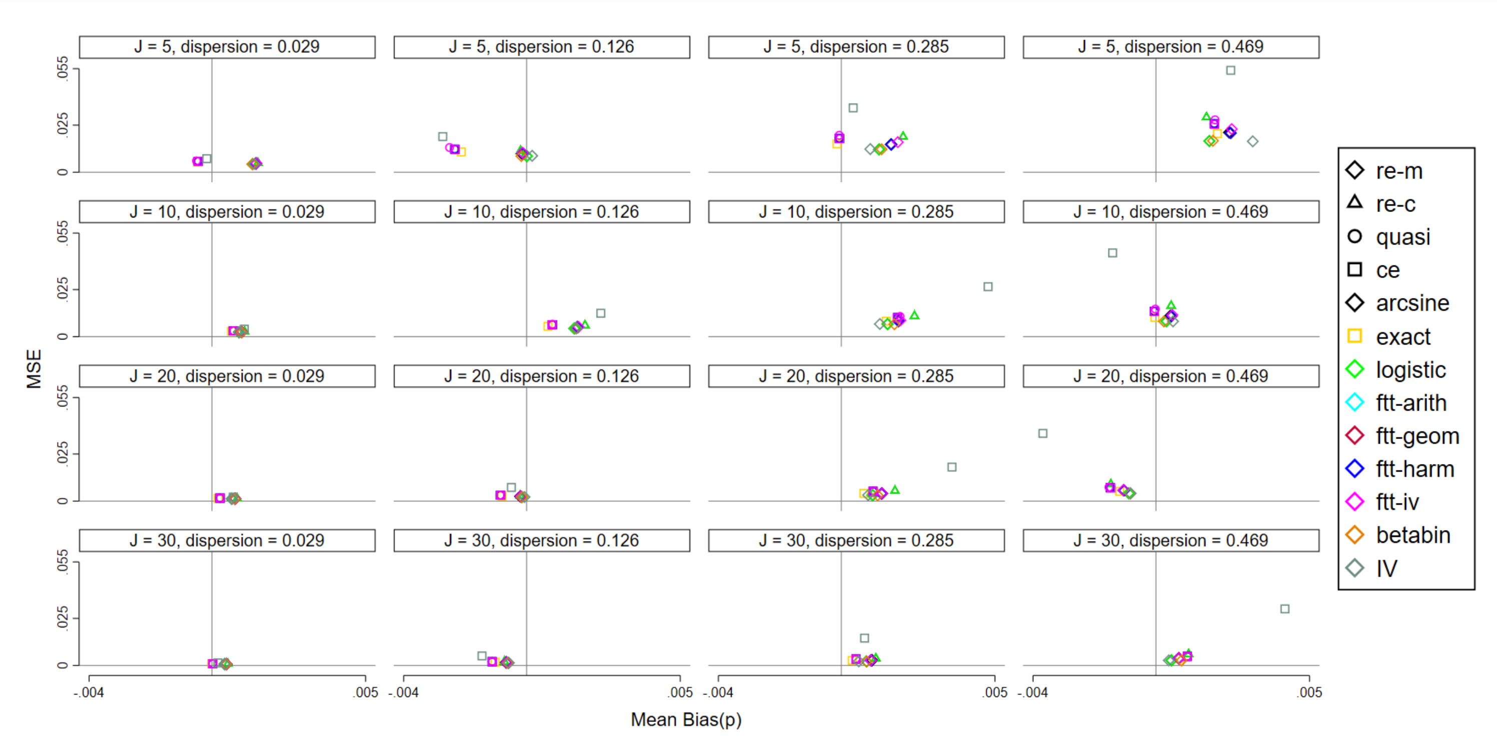

Figure 6: Simulation study. Scatter plot of mean squared error and mean bias of π. Simulated data generated from binomial distribution with $\pi = 0.5$, $J = 5, 10, 20, 30$ and $\tau^2 = [\frac{1}{12}, \frac{1}{16}, \frac{1}{24}, \frac{1}{48}]$. The vertical lines indicate the true population value $\pi = 0.5$.

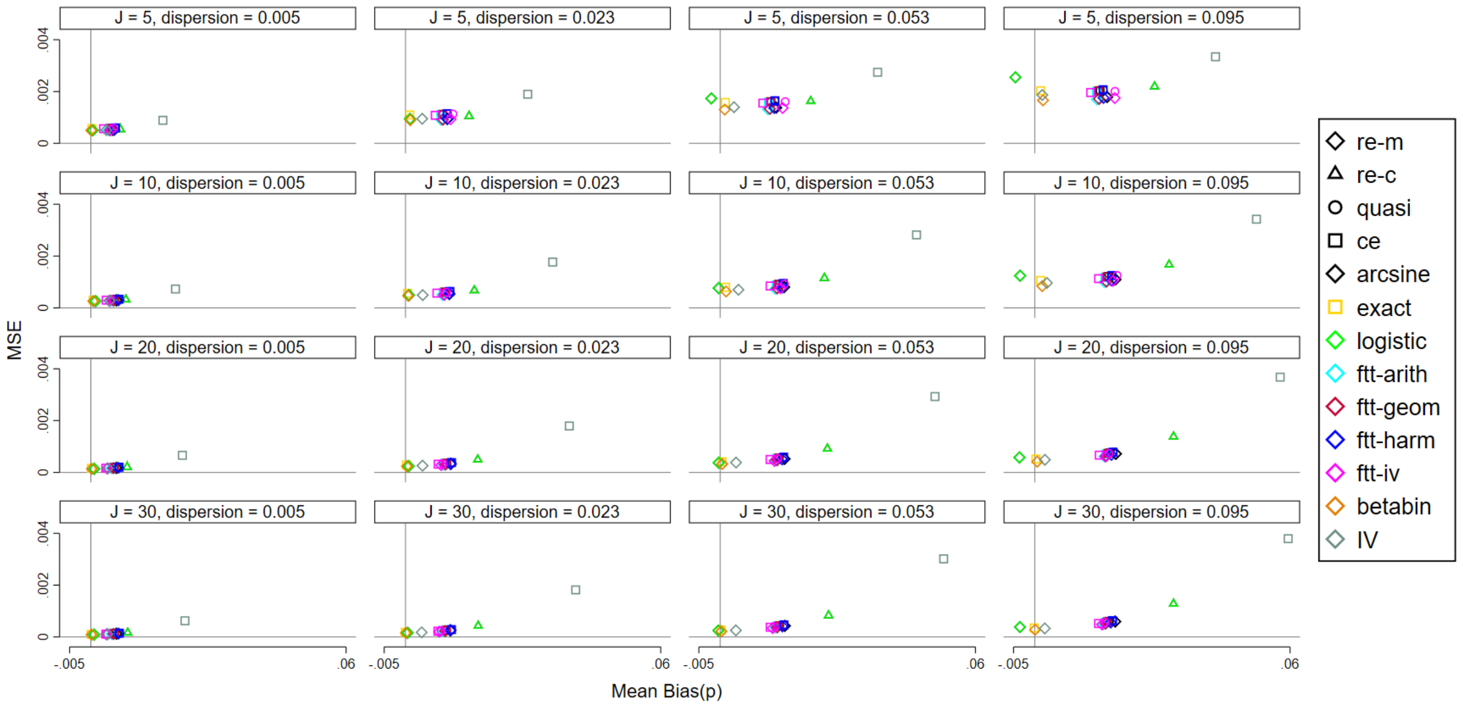

Figure 7: Simulation study. Scatter plot of mean squared error and mean bias of π. Simulated data generated from binomial distribution with $\pi = 0.9$, $J = 5, 10, 20, 30$ and $\tau^2 = [\frac{1}{123}, \frac{1}{164}, \frac{1}{246}, \frac{1}{492}]$. The vertical lines indicate the true population value $\pi = 0.9$.
